# Synthesis and mechanisms of action of novel harmine derivatives as potential antitumor agents

**DOI:** 10.1038/srep33204

**Published:** 2016-09-14

**Authors:** Xiao-Fei Zhang, Rong-qin Sun, Yi-fan Jia, Qing Chen, Rong-Fu Tu, Ke-ke Li, Xiao-Dong Zhang, Run-Lei Du, Ri-hui Cao

**Affiliations:** 1 School of Chemistry, Sun Yat-sen University, Guangzhou, 510275, P. R. China; 2 College of Life Sciences, Wuhan University, Wuhan, 430072, P. R. China; 3 School of Chemistry, Chemical Engineering and Life Sciences, Wuhan University of Technology, Wuhan, 430070, P. R. China; 4 Renmin Hospital of Wuhan University, Hubei General Hospital, Wuhan, 430072, P. R. China

## Abstract

A series of novel harmine derivatives bearing a benzylindine substituent in position-1 of β-carboline ring were synthesized and evaluated as antitumor agents. The N2-benzylated β-carboline derivatives 3a–g represented the most interesting anticancer activities and compound 3c was found to be the most active agent to diverse cancer cell lines such as gastric carcinoma, melanoma and colorectal cancer. Notably, compound 3c showed low toxicity to normal cells. The treatment significantly induced cell apoptosis. Mechanistically, PI3K/AKT signaling pathway mediated compound 3c-induced apoptosis. Compound 3c inhibited phosphorylation of AKT and promoted the production of reactive oxygen species (ROS). The ROS scavenger, LNAC and GSH, could disturb the effect of compound 3c induced apoptosis and PI3K activity inhibitor LY294002 synergistically enhanced compound 3c efficacy. Moreover, the results from nude mice xenograft model showed that compound 3c treatment effectively inhibited tumor growth and decreased tumor weight. Collectively, our results demonstrated that compound 3c exerts apoptotic effect in cancer cells via suppression of phosphorylated AKT and evocation of ROS generation, which suggested that compound 3c might be served as a promising therapeutic agent for cancer treatment.

Cancer is the second leading cause of death in the United States, and is expected to surpass heart diseases as the leading cause of death in the next few years^1^. Intensive research demonstrates that natural product and/or natural product structures continued to play a highly significant role in the drug discovery and development process^2^. β-carboline and its saturated analogue are common structural motifs in natural products and pharmaceuticals^3^. β-carboline alkaloids were originally isolated from Peganum harmala, which have been used as a traditional herbal drug for hundreds of years in cancer in Northwest China^4^.

Harmine (Fig. 1A) is a representative naturally occurring β-carboline alkaloid. Many previous studies were focused on the neuropharmacological effects of harmine on the central nervous system (CNS) such as hallucination, anxiolytic and sedation. Recent interest in harmine has been attracted to its structural modification, structure-activity relationships and mechanisms of action as potential antitumor agents. Isida *et al*.^5^. reported that harmine and β-carboline analogues showed potent cytotoxicity *in vitro* and α-(4-nitrostyryl)-7-methoxy-β-carboline (Fig. 1A) was found to be the most potent antitumor agent. Structure-activity relationships (SARs) analysis revealed that (1) introducing alkoxy substituent into position-7 of harmine led to enhanced cytotoxic activities; (2) the length of alkoxy chain affected both cytotoxicity and cell line specificity; (3) *N*^*9*^-alkylated harmine derivatives exhibited strong cytotoxic effects; (4) *N*^*2*^-alkylated β-carboline derivatives displayed specific cytotoxic activities. Our group^6^ also reported that *N*^*9*^-alkyl and aryl alkyl substituted harmine derivatives exhibited good antitumor activities in mice bearing both Lewis lung carcinoma and Sarcoma 180, while exhibited remarkable neurotoxic effects including tremor, twitch and jumping in experimental animal models. SARs studies suggested that (1) the introduction of appropriate substituents into position- 9 of harmine remarkably enhanced the antitumor activities *in vitro* and *in vivo*; (2) the methoxy group at position-7 of harmine might play a very crucial role in determining their remarkable neurotoxic effects. More recently, our group investigation^7^ on the syntheses of harmine derivatives bearing various substituents at position-2, 7 and 9 of β-carboline nucleus and the evaluation of their antitumor activities *in vitro* and *in vivo* disclosed that (1) the replacement of 7-methoxy group with bulky alkoxy substituent resulted in significant reduce or even elimination neurotoxic effects of harmine; (2) the *N*^*2*^-benzyl substituent on the β-carboline ring played an important role in the modulation of the cytotoxic activities.

The individual harmine derivatives bearing various substituents at different position have been shown to bind to different targets leading to various pharmacological effects. Studies in the past decade revealed that harmine and its analogs exerted their antitumor effects through multiple mechanisms of action including intercalation into DNA^8^,^9^,^10^, inhibition of topoisomerase I (Topo I)^11^,^12^, inhibition cyclin-dependent kinases (CDKs)^13^,^14^, induction cell apoptosis (programmed cell death, PCD) through mitochondrial signaling pathway^15^. In addition, Dai *et al*. reported that harmine activated p53 and subsequently caused inhibition of angiogenesis and tumor growth^16^. Our previous investigations also indicated that harmine and its derivatives induced apoptosis in HepG2 cells and down-regulate the expression of *Bcl-2* gene and upregulate the expression of death receptor *Fas* without altering the level of *Bax* and p53^4^. Recently, JKA97, a benzilydene analogue of harmine (Fig. 1A), was found to induce cell apoptosis and arrest cell in G_0_/G_1_ phase via a p53-independent pathway in human colorectal and breast cancer^17^,^18^.

In a continuing effort to develop novel harmine derivatives endowed with better pharmacological profiles, a series of novel harmine derivatives bearing a benzylindine substituent in position-1 of β-carboline ring were designed and synthesized based on the previously developed SARs. Our investigation demonstrated that all *N*^*2*^-benzylated quaternary β-carbolines had a significant cytotoxic effect against a panel of tumor cell lines, in which compound 3c was found to be the most active agent with IC50 value of 0.46 μM, 0.68 μM and 0.93 μM against BGC-823, A375 and KB cell lines, respectively. In this study, we aimed to screen a highly effective broad spectrum anticancer agent and investigate its mode-of-action.

## Results

### Synthesis

The synthesis of β-carbolines 1a–d has been described in our previous reports^6^,^7^. 1-Benzylidine substituted β-carbolines 2a–f and 2h–j were readily prepared by reaction of 1-methyl-β-carbolines 1a–d with the corresponding aromatic aldehyde in refluxing acetic anhydride^19^. Unexpectedly, the reaction of 1-methyl-β-carboline1a with 4-nitrobenzaldehyde in refluxing acetic anhydride gave *N*^*9*^-acetylated β-carboline 2g. The N^2^-benzylated β-carbolinium bromate derivatives 3a–g was prepared from compounds 2a, 2d–g and 2i–j by the addition of benzyl bromide in refluxing ethyl acetate^19^. The chemical structures of all the newly synthesized compounds were characterized by MS, HRMS, ^1^H NMR and ^13^C NMR.

### Anticancer activity and structure-activity relationship studies

**Figure 1:**
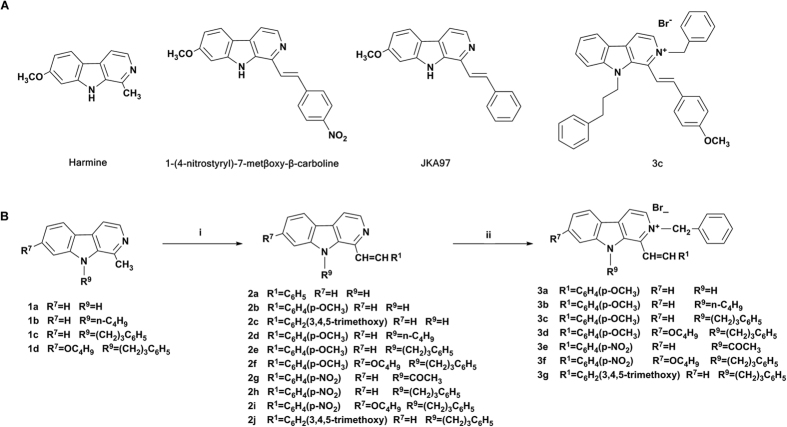
Chemical structures of harmine-based molecules and synthesis scheme of new compounds. (**A**) The structures of harmine and the representative reported (1-(4-nitrostyryl)-7-methoxy-β-carboline and JKA97) and newly synthesized (3c) benzenlidene substituted β-carbolines. (**B**) Reagents and conditions for the synthesis of compounds: (i) Acetic anhydride, R^1^CHO, reflux; (ii) ethyl acetate/benzyl bromide, reflux.

The anticancer potencies of all 17 synthesized compounds (Fig. 1B) against a panel of human tumor cell lines were investigated and compared with the reference drugs cisplatin. The tumor cell line panel consisted of cervical carcinoma (Hela), liver carcinoma (Bel-7402 and HepG2), gastric carcinoma (BGC-823), non-small cell lung carcinoma (A549), malignant melanoma (A375), colon carcinoma (HT-29), renal carcinoma (769-P.OS-RC-2 and 786-0), epidermoid carcinoma of the nasopharynx (KB) and colorectal carcinoma (DLD, HCT116 and RKO). The results were summarized in Table 1.

As shown in Table 1, compounds 2a–c with no substituents at position-9 exhibited moderate cytotoxic activities, but compound 2c, bearing a 3,4,5-trimethoxybenzylidine substituent at position-1, was more active. In our previous investigation, we found that introducing an n-butyl or phenylpropyl substituent into position-9 of β-carboline nucleus facilitated antitumor activities *in vitro* and *in vivo*^6^,^7^. Unfortunately, in the present investigation, compounds 2e–f and 2h–I were almost inactive to all tumor cell lines investigated at the concentration of 200 μM.

The N^2^-benzylated β-carboline derivatives 3a–g represented the most interesting anticancer activities. As predicted, the IC_50_ values of these compounds were lower than 10 μM against most of human tumor cell lines investigated. Compound 3c was found to be the most active agent with IC50 value of 0.46 μM, 0.68 μM and 0.93 μM against BGC-823, A375 and KB cell lines, respectively. Beside, cell lines, such as HepG2 and DLD1, also had lower IC50 values compared with that for other compound 3 (Table 1).

### Compound 3c inhibited cell viability and proliferation of cancer cells

**Figure 2:**
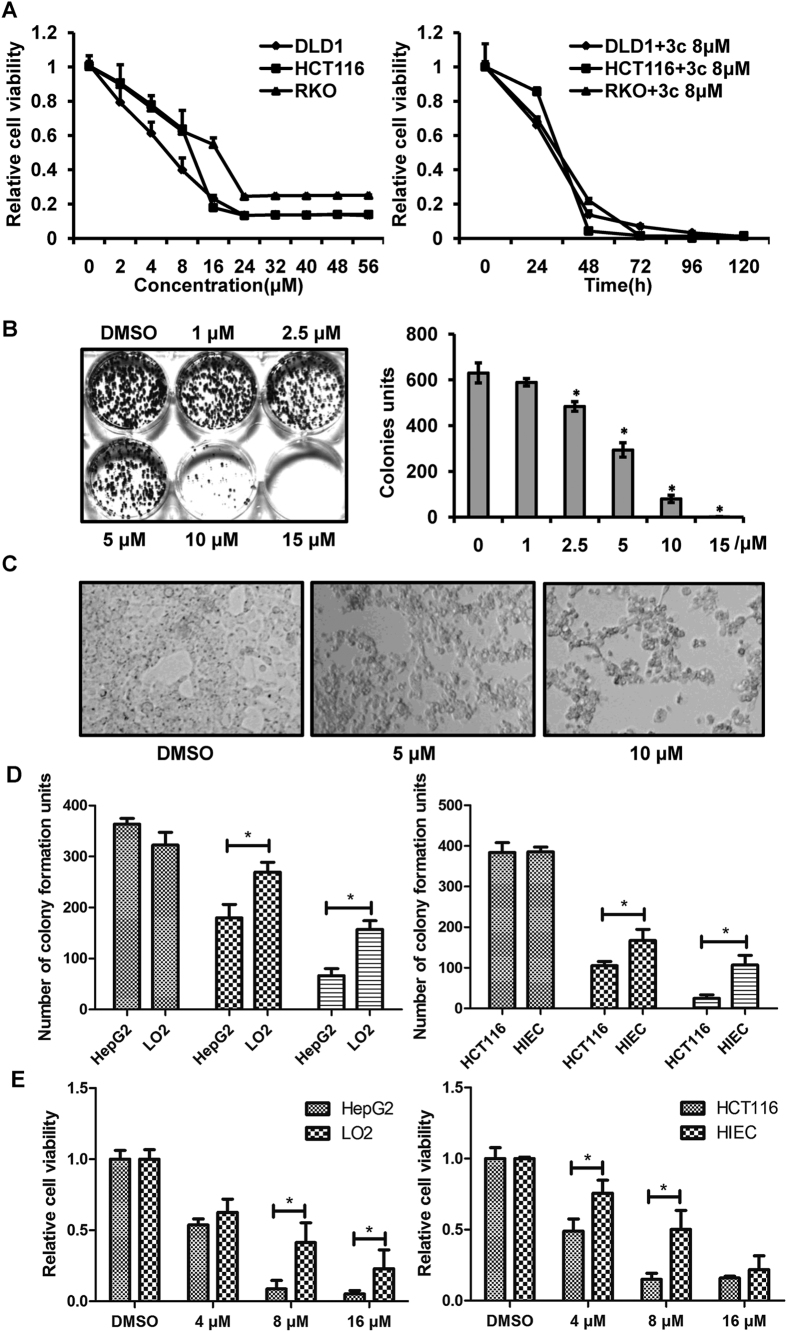
Inhibitory role of compound 3c in cancer cells. (**A**) Three human colorectal cancer cell lines, DLD1, HCT116 and RKO, were seeded in 96 well plates and treated with the indicated concentration of compound 3c for 24 h (left panel) or with the indicated time at the concentration of 8 μM (right panel). Cell viability was determined by CCK8 assays. (**B**) The number of colonies was counted after the treatment of HCT116 cells with the indicated concentration of compound 3c for 24 h. (n = 3, *P < 0.05 versus DMSO control). (**C**) Microphotographs of HCT116 cells treated with drug-free media (DMSO) or media containing 5 μM and 10 μM of compound 3c for 24 h. (**D,E**) Colony formation (left panel) and CCK8 (right panel) assays to determine drug effect on cancer and normal cells, HepG2/LO2 cells in D and HCT116/HIEC cells in E, respectively. Cells seeded in 6 well plates for colony formation assay or 96 well plates for CCK8 assay were treated with compound 3c at indicated concentration. For colony formation assay, n = 3, *P < 0.05 versus normal cells under same condition. For CCK8 assay, n = 6, *P < 0.05 versus normal cells under same condition. The data represents the means ± SDs.

Because compound 3c had a remarkable broad spectrum activity against all tested human cancer cell lines, it was chosen to be further investigated regarding its mechanism of action. We selected human colorectal cell lines as cell model, as compound 3c exhibited diverse cell toxicity to HCT116, RKO and DLD1, the representative cell lines of colorectal cancer which is the second most frequent cause of cancer-related death in the United States^1^. We performed CCK8 and colony formation assay to assess tumor cell growth and proliferation. The results of CCK8 assay showed that compound 3c decreased cell viability of HCT116, RKO and DLD1 in a dose- and time-dependent pathway (Fig. 2A). It was noteworthy that DLD1 was more susceptible to compound 3c when compared with compound 3b and 3f (Supplementary Fig. S1 and Fig. 2A). In HCT116 cells, the colony formation units decreased with the increase of compound 3c concentration (Fig. 2B). Furthermore, phase-contrast micrographs revealed that compound 3c induced cell morphology alteration, such as cell shrinkage and reduced cell number (Fig. 2C). It is clear that there are great difference between metabolism of cancer cells and normal cells. Selective killing tumor cells is a purpose of drug discovery. We analyzed the effect of compound 3c on cancer cells and corresponding normal cells, including HepG2/LO2 (human hepatocyte cells), HCT116/HIEC (human intestinal crypt cells). We found that normal cells were much more resistant to compound 3c with a 1.5~3 fold increase of colony formation units, as shown in Fig. 2D. Cell viabilities of LO2 and HIEC cells were also significantly higher than HepG2 and HCT116 cells at 8 μM compound 3c (Fig. 2E). These data suggested compound 3c as a broad-spectrum anticancer agent with lower cytotoxicity to normal cells compared with corresponding cancer cells.

### Compound 3c induced mitochondria-mediated cell apoptosis in HCT116 cells

**Figure 3:**
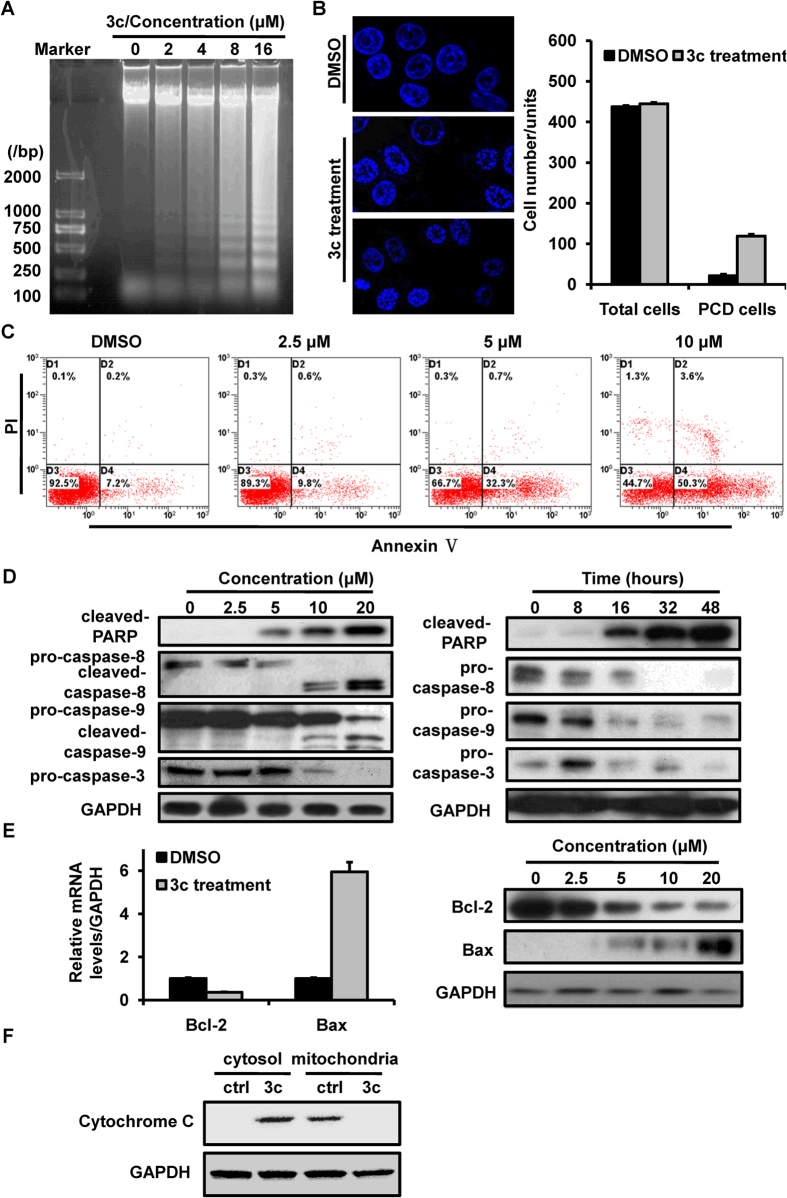
Compound 3c induced cell apoptotic pathway in HCT116 cells. (**A**) DNA fragmentation assay of cell apoptosis in HCT116 cells treated with the indicated concentration of compound 3c for 24 h. (**B**) Confocal microscopy of HCT116 cells treated with 8 μM of compound 3c. The non-treated and treated cells were stained with DAPI and observed by confocal microscopy (left panel). The right panel showed the statistics of apoptotic cells in several random fields of the staining images. (*P < 0.05 versus DMSO control) (**C**) HCT116 cells were treated with the indicated concentration of compound 3c for 24 h and subjected into flow cytometry analysis. (**D**) HCT116 cells were treated with the indicated concentration of compound 3c for 24 h (left panel) or with the indicated time at the concentration of 8 μM (right panel). The levels of apoptotsis-associated proteins, including PARP, caspase-8, caspase-9 and caspase-3 were detected by western blotting analysis. (**E**) The mRNA (left panel) and protein (right panel) alteration of Bcl-2 and Bax in HCT116 cells treated with 8 μM of compound 3c. (**F**) The levels of Cytochrome C in cytosol and mitochondria. HCT116 cells were treated with 8 μM of compound 3c for 24 h and the cytosolic and mitochondria proteins were isolated by density gradient centrifugation. GAPDH was used as an internal control. The data represents the means ± SDs.

In response to overwhelming external stimuli, cells are forced to die in different ways. Cells are prone to apoptosis, a programmed cell death through the activation of caspase cascade^20^. The apoptotic-positive cells often have morphological features such as cell shrinkage, chromatin condensation or nuclear fragmentation. Here, we surveyed chromatin pyknosis by a DNA fragmentation assay. The agarose gel electrophoresis showed a DNA fragmentation in HCT116 cells as early as 4 hours after compound 3c treatment (Fig. 3A). Then we treated HCT116 cells with 8 uM compound 3c and stained with DAPI. The images from a confocal laser-scanning microscope expressed the morphological alterations of cell nucleus. The apoptotic cells (PCD cells) were numbered, and the apoptotic rate was about 25.8% (115 of 445) in cells treated with compound 3c, but only 4.09% (18 of 440) in cells treated with DMSO (Fig. 3B). Next, the treated or non-treated cells were subjected to FACS cytometry. Under DMSO conditions, <8% of HCT116 cells were positive for Annexin V. When the compound 3c concentration was up to 5 μM, more than 30% cells were stained positively for Annexin V. 50.3% cells entered the apoptotic pathway as the compound 3c concentration was up to 10 μM (Fig. 3C).

The cleavage of PARP and caspases was the marker of cell apoptosis. Western blotting analysis was used to detect the levels of apoptosis-related protein. As shown in Fig. 3D, compound 3c treatment of HCT116 cells led to an effective induction of the cleavage of PARP, caspase-8, caspase-9 and caspase-3 accompanied with an obvious decrease of total protein levels of these proteins. Due to the involvement of caspase-8 in extrinsic apoptosis pathway and caspase-9 in intrinsic apoptosis pathway, our results suggested that both extrinsic and intrinsic apoptosis pathway were participated in compound 3c-induced cancer cell death. Pro-apoptotic protein Bax and anti-apoptotic protein Bcl-2, the members of the Bcl-2 family, plays a pivotal role in mitochondria pathway of apoptosis. We detected the mRNA and protein levels of Bax and Bcl-2 and observed an increase of Bax, and a decrease of Bcl-2 in compound 3c treated cells (Fig. 3E).

Furthermore, we investigated the release of cytochrome c from the mitochondria to the cytosol, a downstream apoptotic event in mitochondria-mediated apoptosis. Western blotting analysis showed that 8 μM compound 3c treatment for 24 hours led to an accumulation of cytochrome c in the cytosol and a decrease in the mitochondria (Fig. 3F). These results from our study demonstrated that compound 3c treatment induced the mitochondria mediated cytochrome c release and caspase activation.

### Compound 3c-induced cell apotosis was via suppression of PI3K/AKT signaling pathway

**Figure 4:**
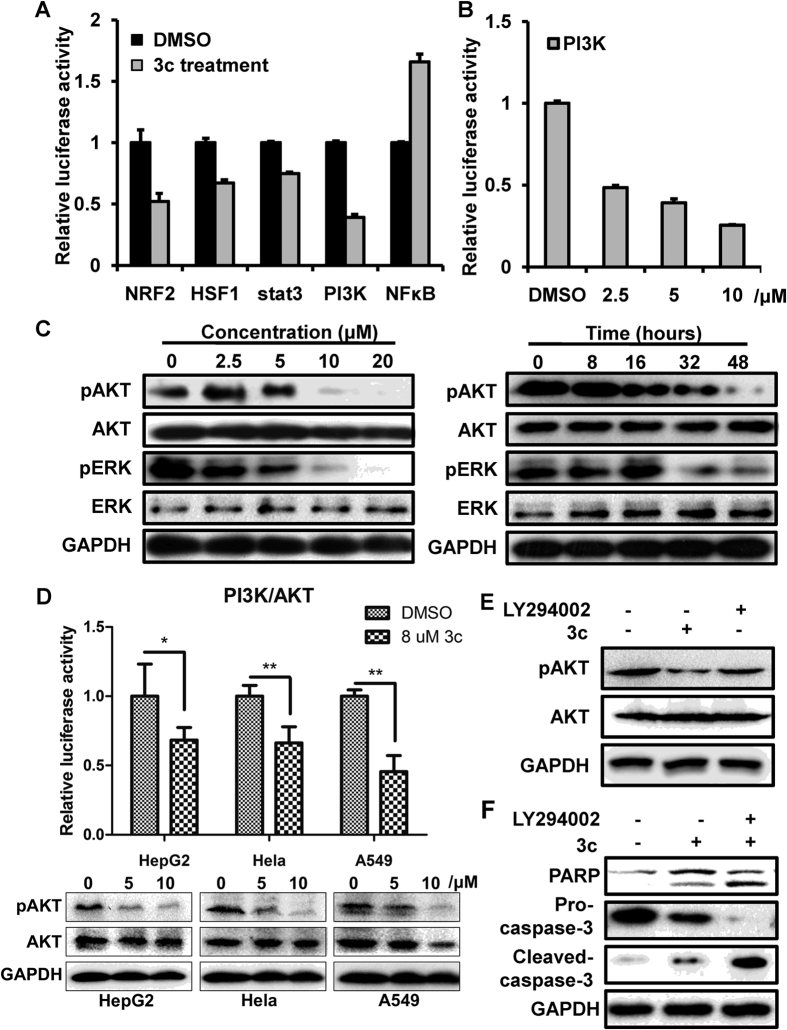
Compound 3c induced cell apoptosis through suppression of PI3K/AKT signaling pathway in cancer cells. (**A**) Luciferase activity of NRF2, HSF1, stat3, PI3K and NFκB signaling pathway reporter. HCT116 cells were transfected with the indicated pathway reporter for 24 h, treated with 4 μM of compound 3c for another 24 h and then subjected to dual-luciferase assay. (**B**) Dose-dependent reduction of PI3K reporter activity. The cells were incubated with the indicated concentration of compound 3c for 24 hours. (**C**) Dose- and time- dependent inhibition of the levels of phosphorylated AKT and ERK. HCT116 cells were treated with the indicated concentration of compound 3c for 48 hours or with 8 μM compound 3c for 8, 16, 32 and 48 hours. (**D**) Luciferase analysis of PI3K/AKT reporter activities and western blotting for measurment of pAKT levels in HepG2, Hela and A549. For luciferase assay, cells were treated with 8 μM compound 3c for 18 hours. (n = 3, *P < 0.05 versus DMSO). For western blotting, cells were treated with the indicated concentration of compound 3c for 72 h. (**E**) LY294002 inhibited the phosphorylation level of AKT. HCT116 cells were treated with 8 μM compound 3c alone for 24 hours or in combination with pretreatment with 50 μM LY294002 for 4 hours. (**F**) The protein levels of PARP, caspase-3 and cleaved caspase-3 in HCT116 cells treated with 8 μM compound 3c alone or in combination with 50 μM LY294002 for 4 h. The data represents the means ± SDs. GAPDH was used as an internal control.

Numerous signaling pathways are involved in regulation of cell survival. To elucidate the pathway or process regulated by compound 3c, we screened the 45 signaling pathways purchased from Qiagen using luciferase assay in HCT116 cells. The results demonstrated that the luciferase activities of several pathways were altered after compound 3c treatment, including PI3K, NF-kappa B, stat3 and HSF1 (Supplementary Fig. S2). Notably, the activity of PI3K was reduced by about 60% (Fig. 4A). We performed a dose-response experiment and confirmed the effect of compound 3c on the activity of PI3K (Fig. 4B). Western blotting analysis was used to evaluate the activity of PI3K related protein kinases after compound 3c treatment. We found that the phosphorylated levels of AKT and ERK were decreased after compound 3c treatment and the total protein levels of AKT and ERK stayed the same, and this process was dose- and time- dependent (Fig. 4C). In order to determine whether PI3K/AKT inactivation was an extensive phenomena in various tumor cells, we analyzed PI3K/AKT reporter activities and levels of phosphorylated AKT in HepG2, A549 and Hela cell lines. Results showed that PI3K/AKT activities were decreased about 32%, 34% and 54% in HepG2, A549 and Hela cells, respectively. The levels of pAKT were reduced consistently (Fig. 4D). The above data demonstrated that compound 3c induced cell apoptosis through suppression of PI3K/AKT signaling in various cancer cells.

The above results indicated that compound 3c suppressed PI3K/AKT signaling. LY294002, a chemical inhibitor of PI3K, was shown to exert additive and synergistic effects when used in combination with anti-cancer agents^21^,^22^. HCT116 cells were exposed to 8 μM compound 3c with or without LY294002. We found that AKT activation was blocked by LY294002 and compound 3c, and the effect of latter was more apparent (Fig. 4E). To further investigate the function of LY294002 in compound 3c induced apoptosis, we detected the levels of cleavaged PARP and caspase-3 and found a significant increase of cleaved proteins in cells treated with 8 μM compound 3c in combination with pretreatment of 50 μM LY294002 (Fig. 4F). These data indicated that LY294002 synergistically enhanced the efficacy of compound 3c and suggested compound 3c as a more potent chemotherapeutic agent when combined with PI3K inhibitor.

### ROS accumulation was involved in compound 3c-induced cell apoptosis

**Figure 5:**
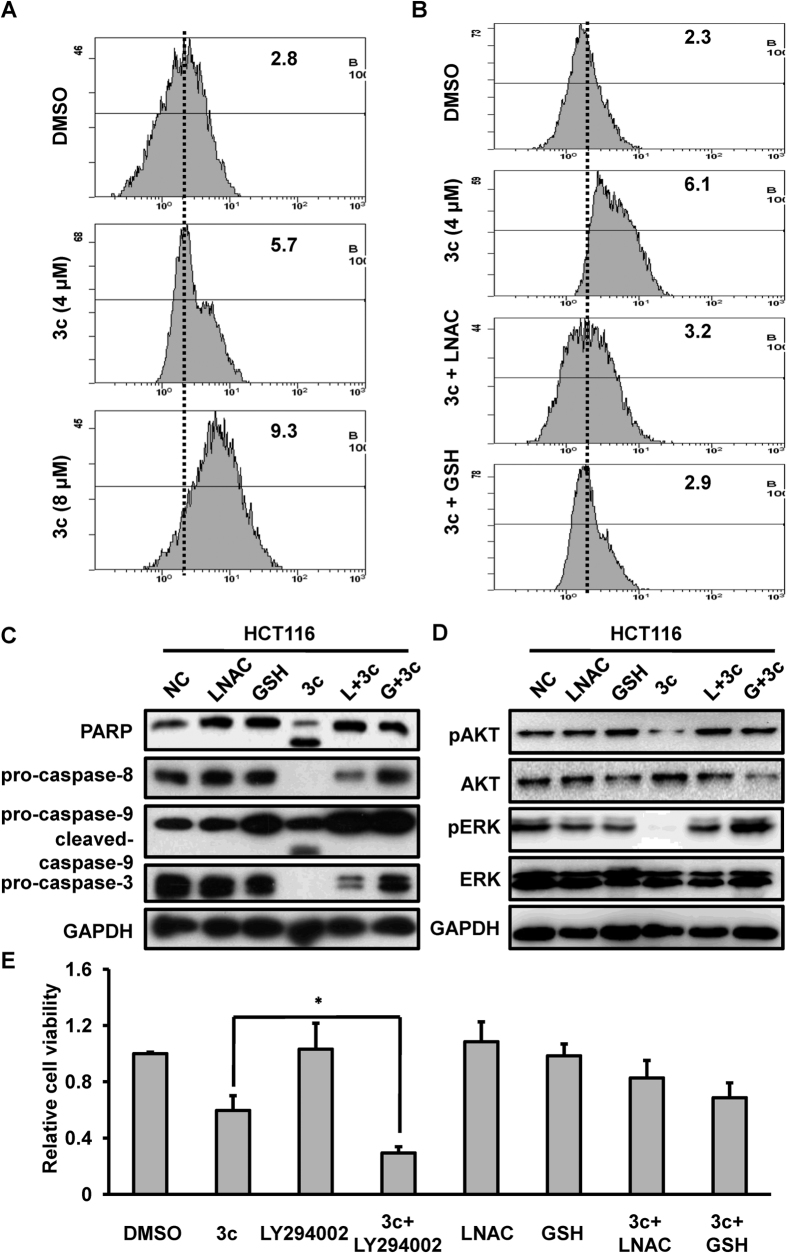
Compound 3c promoted ROS generation in HCT116 cells. For flow cytometry analysis, HCT116 cells were treated with the indicated concentration of compound 3c for 24 hours alone or in combination with pretreatment of 20 mM LNAC or 20 mM GSH for 2 hours. For western blotting and cell viability assay, HCT116 cells were pretreated with 20 mM LNAC, 20 mM GSH or 50 μM LY294002 for 2 hours, and then treated with 8 μM compound 3c for another 24 hours. (**A**) Compound 3c increased the levels of ROS in HCT116 cells. (**B**) Pretreatment with LNAC and GSH attenuated the production of ROS resulted from compound 3c treatment. (**C,D**) Pretreatment with LNAC and GSH blocked the cleavage of PARP, caspase-8, caspase-9 and caspase-3 and partially restored the phosphorylated levels of AKT and ERK. (**E**) The cell viability of HCT116 cells was determined by CCK8 assays after treatment with compound 3c or in combination with LY294002/LNAC/GSH and the corresponding control. *P < 0.05 compared with cells treated with compound 3c. The data represents the means ± SDs. GAPDH was used as an internal control.

ROS are essential for various biological processes in normal cells; however, the excessive production of ROS kills cancer cells. Many anticancer drugs initially induce ROS generation and kill cancer cells through apoptotic pathway^23^. In this study, using the CM-H_2_DCFDA fluorescent as a ROS indicator, we observed an increase of ROS production in a dose-dependent manner after compound 3c treatment (Fig. 5A). To further explore the possibility of ROS as an initiator of compound 3c-induced cell apoptosis, we pretreated HCT116 cells with glutathione (GSH) or L- N-acetyl-cysteine (LNAC) for 2 hours, followed by treatment with 8 μM compound 3c for another 24 hours. To remove the possibility of the chemical reaction between LNAC/GSH and compound 3c, we firstly performed the chemical reaction of compound 3c with various thiols and failed to detect the formation of new product. FACS analysis showed that GSH and LNAC reduced compound 3c -induced ROS accumulation from 6.1 to 3.2 and 2.9, respectively (Fig. 5B). Western blotting analysis showed that the removal of ROS by GSH or LNAC reversed compound 3c-induced cell apoptosis, indicated by decreased cleavage of PARP and caspase-3 (Fig. 5C).

Furthermore, in compound 3c treated HCT116 cells, the phosphorylated levels of AKT and ERK were increased following GSH or LNAC pretreatment (Fig. 5D). We also assayed cell viability of cells treated with compound 3c or compound 3c in combination with LY294002. The result of CCK8 assay showed that the cell viability of HCT116 was decreased in cells treated with compound 3c in combination with LY294002, compared with cells treated with compound 3c alone (Fig. 5E). Therefore, our study illustrated that compound 3c induced ROS accumulation and subsequently induced PI3K/AKT inactivation and triggered cell apoptosis.

### Compound 3c inhibited tumorigenecity of colorectal cancer cell line HCT116 in nude mice

**Figure 6:**
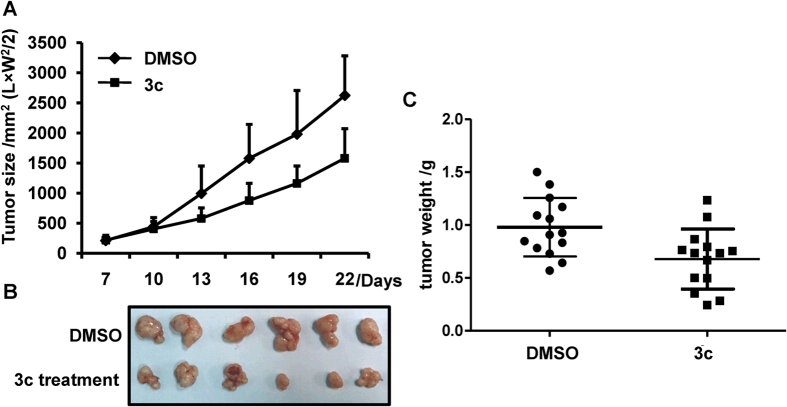
Compound 3c inhibited tumor growth *in vivo*. HCT116 cells were injected into mice to establish *in vivo* tumor model according to the instructions in the Materials and Methods. (**A**) The tumor size was measured every three days. (**B**) The tumors were removed from mice after 21 days growth. (**C**) The removed tumors were weighted and statistically analyzed. (n = 10) The data represents the means ± SDs.

To further examine the anticancer role of compound 3c *in vivo*, we established human colorectal cancer xenograft model in 6-week-old female nude mice by subcutaneously injecting HCT116 cells (5 × 10^6^) into the flanks of the mice. When the tumors grew to 3–5 mm in diameter, a total of 20 mice were divided randomly into two groups, a vehicle control group and a compound 3c treatment group. Compound 3c (50 mg/Kg body weight) was administered once every three day for 21 days, and the tumor sizes were measured every three days. Compound 3c treatment decreased tumor volume at day 13 as compared with the DMSO control group, confirming the anticancer activity of compound 3c *in vivo* (Fig. 6A). The tumors isolated from mice were photographed and there was an overt smaller in group treated with compound 3c (Fig. 6B). The tumor weight of the control group was apparently higher than compound 3c treatment group (Fig. 6C). These results indicated that compound 3c exhibits antitumor activity *in vivo* by induction of cell apoptosis.

## Discussion

In the present investigation, a series of novel harmine derivatives bearing a benzylindine substituent in position-1 of β-carboline ring were synthesized and evaluated as antitumor agents. The N^2^-benzylated β-carboline derivatives 3a–g represented the most interesting anticancer activities and compound 3c was found to be the most active derivative with IC_50_ value of 0.46 μM, 0.68 μM, and 0.93 μM against BGC-823, A375, and KB cell lines, respectively. Current investigation corroborated our previous observations that (1) introducing appropriate substituents into position-9 of β-carboline nucleus enhanced their cytotoxic activities; (2) the *N*^*2*^-benzyl substituent on the β-carboline core played a very important role in the modulation of the cytotoxic potencies.

Some important molecular mechanisms of action of harmine as antitumor agent have recently been reviewed^19^. Previous studies demonstrated that harmine could inhibit cell proliferation and induce cell apoptosis by activation of p53 signaling pathway. Recent investigation reported that harmine derivatives triggered anticancer roles through p53-independent pathway. So the exact mechanism of how harmine and its derivatives work in various tumors was not fully understood. We aimed to screen a highly effective broad spectrum anticancer derivative and investigate its mode-of-action in cancers. Compound 3c were selected for further analysis because its broad spectrum high cytotoxicity to various tumor cell lines (Supplementary Fig. 1 and Fig. 4B). Our investigation indicated that compound 3c effectively inhibited cancer cells growth at the low concentration and subsequently led the cells to enter the apoptotic pathway which was indicated by cleavage of caspase 9, caspase 8, caspase 3 and PARP. The characteristics of cell apoptosis, including DNA fragmentation and chromatin condensation, were also observed in HCT116 cells treated with compound 3c. More importantly, normal cells exhibited significantly greater resistance to compound 3c compared with cancer cells.

Mitochondria are the parts of a central mechanism of amplification of the apoptotic signal. The loss of integrity of the outer mitochondrial membrane caused by pro-apoptotic members of the Bcl-2 family resulted in the release of cytochrome c from mitochondria to the cytosol and subsequently to promote caspase activation^24^,^25^. In this study, we found that compound 3c induced cell apoptotic pathway through a mitochondria-dependent manner. Pro-apoptotic protein Bax was up-regulated and anti-apoptotic Bcl-2 was down-regulated following compound 3c treatment. In addition, compound 3c led to the release of cytochrome c to cytosol. Mitochondria also play the essential role to supply the cells with metabolic energy in the form of ATP, the primary source of cellular ROS, which makes them be the major sites of ROS production^26^. It was widely accepted that ROS activation was associated with the cell apoptotic pathway induced by various anticancer drugs^27^,^28^. We also observed an accumulation of ROS in cells treated with compound 3c. Antioxidants such as GSH and LNAC pretreatment could partially block compound 3c induced cell apoptosis. Therefore, compound 3c induced cell apoptosis was largely mediated and controlled by mitochondria signaling pathway.

Reactive oxygen species (ROS) have been reported to be involved in numerous key cellular processes^29^. Cancer cells have higher levels of ROS than normal cells and increased ROS levels at the site of carcinogenesis are linked to the accelerated formation of metastasis^30^. Overproduction of ROS resulted from radiation and anticancer agents were related with cellular damage^31^,^32^. In clinic, commonly used anticancer agents such as anthracyclines and cisplatin induce the generation of ROS in cells, which mediated drug-induced cell apoptosis. ROS function as messengers in signaling transduction by activating various signaling pathway, including MAPK, p38, AKT and signal transducers and activators of transcription^33^. We found that compound 3c induced cell apoptosis in human colorectal cancer cell lines, and this process was mediated by accumulation of ROS and inactivation of PI3K/AKT signaling pathway. The levels of pAKT were up-regulated in cells following ROS removal by GSH or LNAC. Thus, our data suggested that ROS production might be an upstream event leading to PI3K/AKT inactivation during compound 3c induced cell apoptosis. Notably, in our study, we found that compound 3c also altered HSF1, Nrf2 and NF-κb signaling activities. Given the requirement of ROS accumulation in compound 3c function, we were thinking that ROS activation might modulate the activities of above signaling reporters. H_2_O_2_, an intracellular ROS inducer, was used to treat HCT116 cells and we obtained a similar results to compound 3c treated cells (Supplementary Fig. 3). These data further confirm the upstream effect of ROS accumulation.

In response to different cell death stimuli, multiple signaling pathways were activated or inactivated to modulate the survival of cancer cells. It is well known that PI3K/AKT signaling pathway is crucial in promotion of cell growth, survival, and metabolism. Activated PI3K in cells plays an important role in inhibition of cell apoptosis^34^. Therapeutics targeting the PI3K pathway was being developed at a rapid pace^35^. In drug discovery, the action of anticancer agents is critical in clinic, for example, the unique mechanism of an agent is urgent and practical for guidance of multi-drug combined chemotherapy for various tumors^36^. We screened PI3K/AKT signaling pathway as a key mediator involved in compound 3c function. Compound 3c decreased the phosphorylation of AKT in various cancers, including breast cancer, colorectal cancer, hepatocellular carcinoma and lung cancer. AKT inhibitor LY294002 could synergistically promote cell apoptosis in colorectal cancer. The results collectively supported compound 3c as an effective agent with unique mode-of-action in cancer therapy, and also provided evidence for the LY294002 combination therapy. It was worth mentioning that compound 3b showed weaker inhibition effect to PI3K/AKT activities in HCT116 when compared with compound 3c despite the higher cytotoxicity of compound 3b to HCT116 (Supplementary Fig. 4A,B). In addition, the cytotoxicities of compound 3b were greatly different in various tumor cell lines, which indicated its cell context dependent effectiveness (Supplementary Fig. 4C). These observations also suggested distinct function mechanisms of individual N^2^-benzylated β-carboline derivatives.

Our *in vivo* experiments displayed a tumor inhibition effect of compound 3c. However, we only analyzed the inhibition effect of compound 3c on HCT116 cells in xenograft model, its effects on other cancers and LY294002 combination effects need further investigation.

In summary, compound 3c, a novel drug synthesized based on harmine, functioned as an anticancer agent with a low toxicity. The induction of apoptosis effect was accompanied at a lower concentration of compound 3c. ROS overproduction was an upstream event and mediated the inhibition of AKT phosphorylation; and subsequently activated the mitochondria dependent cell apoptotic pathway. Our study synthesized a new harmine derivative and provided a novel and unique function mechanism of it as a promising antitumor agent for cancer treatment.

## Methods

### General information

All reagents were purchased from commercial suppliers and were dried and purified when necessary, and compounds 1a–d was prepared as previously described^9^,^10^. Melting points were determined in capillary tubes on an electrothermal PIF YRT-3 apparatus and without correction. ESI-MS spectra were obtained from VG ZAB-HS spectrometer. ^1^H NMR and ^13^C NMR spectra were recorded on a AVANCE III 400MHz spectrometer at 400 MHz and 100 MHz and a Varian INOVA 500NB spectrometer at 500 MHz and 125 MHz, respectively, using TMS as internal standard and CDCl_3_ or DMSO-*d*_6_ as solvent and chemical shifts (d) were expressed in ppm. HRMS were obtained from ESI-Q-TOF maxis 4G spectrometer. Silica gel F254 were used in analytical thin-layer chromatography (TLC) and silica gel were used in column chromatography respectively.

### General synthetic procedure for the preparation of 1-benzylidine β-carbolines 2a–j

A mixture of 1a–d (10 mmol), acetic anhydride (50 ml) and aldehydes (50–100 mmol) was refluxed for 24–48 h. After completion of the reaction as indicated by TLC, the solution was poured into ice-water (200 ml) and made basic with sodium bicarbonate. The aqueous mixture was extracted with ethyl acetate, and the organic phase was washed with water and brine and then dried over anhydrous sodium sulfate. The solvent was removed under vacuum, and the residue was dissolved in ethanol and made acidic with concentrated hydrochloric acid. The solvent was evaporated in reduced pressure and the resulting oil was crystallized from acetone to give yellow solid. The solid was dissolved in water and made basic with sodium bicarbonate, and the aqueous mixture was extracted with ethyl acetate. The organic phase was washed with water and brine, dried over anhydrous sodium sulfate, concentrated under vacuum. The oil residue was crystallized to give 2a–j in 12–65% yields.

#### 1-Styryl-β-carboline (2a)

Yellow solid was obtained (0.4 g, 15%). Mp 204–205 °C; ESI-MS m/z: 271 [M+H]^+^; ^1^H NMR (400 MHz, CDCl_3_) *δ* 8.78 (s, 1H, Ar*H*), 8.52 (d, *J* = 5.2 Hz, 1H, Ar*H*), 8.14 (d, *J* = 8.0 Hz, 1H, Ar*H*), 7.91–7.83 (m, 2H, Ar*H*), 7.60–7.52 (m, 5H, Ar*H*, CH = C*H*C_6_H_5_), 7.40–7.26 (m, 4H, Ar*H*, C*H* = CHC_6_H_5_); ^13^C NMR (100 MHz, DMSO-*d*_*6*_) *δ* 141.0, 139.5, 138.8, 137.3, 134.6, 132.0, 129.3, 129.0, 128.7, 127.5, 123.8, 122.3, 121.4, 119.9, 114.2, 112.4; HRMS (ESI) calcd for C_19_H_14_N_2_ [M+H]^+^ 271.1230, found 271.1231.

#### 1-(4-Methoxystyryl)-β-carboline (2b)

Yellow solid was obtained (0.36 g, 12%). Mp 215–217 °C; ESI-MS m/z: 301 [M+H]^+^; ^1^H NMR (400 MHz, CDCl_3_) *δ* 8.68 (s, 1H, Ar*H*), 8.49 (d, *J* = 5.2 Hz, 1H, Ar*H*), 8.14 (d, *J* = 8.0Hz, 1H, Ar*H*), 7.86–7.81 (m, 2H, Ar*H*), 7.58–7.52 (m, 4H, Ar*H*, CH = C*H*C_6_H_4_(p-OCH_3_)), 7.40 (d, *J* = 16 Hz, 1H, C*H* = CHC_6_H_4_(p-OCH_3_)), 7.38– 7.29 (m, 1H, Ar*H*), 6.90 (d, *J* = 8.8 Hz, 2H, Ar*H*), 3.83 (s, 3H, OC*H*_3_); ^13^C NMR (100 MHz, DMSO-*d*_*6*_) *δ* 160.0, 140.9, 140.0, 138.8, 134.3, 131.8, 130.0, 128.9, 128.8, 128.6, 122.2, 121.4, 119.8, 114.8, 113.8, 112.3, 55.7; HRMS (ESI) calcd for C_20_H_16_N_2_O [M+H]^+^ 301.1335, found 301.1356.

#### 1-(3,4,5-Trimethoxystyryl)-β-carboline (2c)

Yellow solid was obtained (0.42 g, 12%). Mp 134–135 °C; ESI-MS m/z: 361 [M+H]^+^; ^1^H NMR (400 MHz, CDCl_3_) *δ* 9.25 (s, 1H, Ar*H*), 8.53 (d, *J* = 5.2 Hz, 1H, Ar*H*), 8.15 (d, *J* = 8.0 Hz, 1H, Ar*H*), 7.89 (d, *J *= 5.2 Hz, 1H, Ar*H*), 7.79 (d, *J* = 5.6 Hz, 1H, CH = C*H*C_6_H_2_(m, m, p-OCH_3_)), 7.55 (d, *J* = 6.4 Hz, 2H, Ar*H*), 7.48 (d, *J *= 4.0 Hz, 1H, C*H* = CHC_6_H_2_(m, m, p-OCH_3_)), 7.34–7.30 (m, 1H, Ar*H*), 6.76 (s, 2H, Ar*H*), 3.86 (s, 3H, p-OC*H*_3_), 3.79 (s, 6H, m-OC*H*_3_); ^13^C NMR (100 MHz, DMSO-*d*_*6*_) *δ* 153.3, 140.5, 139.2, 138.4, 138.1, 134.0, 132.5, 132.1, 128.6, 128.4, 122.5, 121.9, 121.0, 119.6, 113.7, 111.9, 104.7, 60.2, 56.1; HRMS (ESI) calcd for C_22_H_20_N_2_O_3_ [M+H]^+^ 361.1547, found 361.1556.

#### 1-(4-Methoxystyryl)-9-butyl-β-carboline (2d)

Yellow solid was obtained (1.6 g, 45%). Mp 250–252 °C; ESI-MS m/z: 357 [M+H]^+^; ^1^H NMR (400 MHz, CDCl_3_) *δ* 8.49 (d, *J* = 4.8 Hz, 1H, Ar*H*), 8.15 (d, *J* = 7.6 Hz, 1H, Ar*H*), 7.89 (d, *J* = 5.2 Hz, 1H, Ar*H*), 7.77 (dd, *J* = 37.6, 15.2 Hz, 2H, C*H* = C*H*C_6_H_4_(p-OCH_3_)), 7.65–7.56 (m, 3H, Ar*H*), 7.50 (d, *J* = 8.4 Hz, 1H, Ar*H*), 7.32 (d, *J* = 7.6 Hz, 1H, Ar*H*), 6.98 (d, *J* = 8.8 Hz, 2H, Ar*H*), 4.60 (t, *J* = 7.6 Hz, 2H, NC*H*_2_CH_2_CH_2_CH_3_), 3.88 (s, 3H, OC*H*_3_), 2.06–1.98 (m, 2H, NCH_2_C*H*_2_CH_2_CH_3_), 1.55–1.46 (m, 2H, NCH_2_CH_2_C*H*_2_CH_3_), 1.01 (t, *J* = 7.2Hz, 3H, NCH_2_CH_2_CH_2_C*H*_3_); ^13^C NMR (100 MHz, CDCl_3_) *δ* 159.4, 141.5, 139.8, 138.3, 133.6, 133.3, 129.8, 129.7, 128.0, 127.9, 121.5, 121.1, 120.8, 119.3, 113.9, 112.9, 109.2, 55.0, 45.0, 32.0, 20.1, 13.6; HRMS (ESI) calcd for C_24_H_24_N_2_O [M+H]^+^ 357.1961, found 357.1953.

#### 1-(4-Methoxystyryl)-9-(3-phenylpropyl)-β-carboline (2e)

Yellow solid was obtained (2.2 g, 53%). Mp 136–137 °C; ESI-MS m/z: 419[M+H]^+^; ^1^H NMR (400 MHz, CDCl_3_) *δ* 8.47 (d, *J* = 4.8 Hz, 1H, Ar*H*), 8.11 (d, *J* = 7.6 Hz, 1H, Ar*H*), 7.86 (d, *J* = 5.2 Hz, 1H, Ar*H*), 7.78 (d, *J* = 15.6 Hz, 1H, CH = C*H*C_6_H_4_(p-OCH_3_)), 7.68 (d, *J* = 15.6 Hz, 1H, C*H* = CHC_6_H_4_(p-OCH_3_)), 7.57–7.53 (m, 3H, Ar*H*), 7.34–7.27 (m, 4H, Ar*H*), 7.22–7.15 (m, 3H, Ar*H*), 6.97–6.93 (m, 2H, Ar*H*), 4.60 (t, *J* = 7.8 Hz, 2H, NC*H*_2_CH_2_CH_2_C_6_H_5_), 3.86 (s, 3H, OC*H*_3_), 2.77 (t, *J* = 7.8 Hz, 2H, NCH_2_CH_2_C*H*_2_C_6_H_5_), 2.36–2.28 (m, 2H, NCH_2_C*H*_2_CH_2_C_6_H_5_); ^13^C NMR (100 MHz, DMSO-*d*_*6*_) *δ* 159.7, 144.1, 140.8, 138.9, 135.2, 133.9, 132.6, 132.0, 130.6, 130.1, 128.3(2C), 126.4, 126.0, 123.6, 121.8, 119.4, 116.8, 115.3, 114.3, 111.4, 55.2, 44.0, 32.0, 31.4. HRMS (ESI) calcd for C_29_H_26_N_6_O [M+H]^+^ 419.2118, found 419.2128.

#### 1-(4-methoxystyryl)-7-butoxy-9-(3-phenylpropyl)-β-carboline (2f)

Yellow solid was obtained (2.7 g, 55%). Mp 110–111 °C; ESI-MS m/z: 491 [M+H]^+^; ^1^H NMR (400 MHz, CDCl_3_) *δ* 8.42 (d, *J* = 4.8 Hz, 1H, Ar*H*), 7.95 (d, *J *= 8.4 Hz, 1H, Ar*H*), 7.77 (d, *J* = 15.2 Hz, 1H, CH = C*H*C_6_H_4_(p-OCH_3_)), 7.75 (d, *J* = 4.8 Hz, 1H, Ar*H*), 7.66 (d, *J* = 15.2 Hz, 1H, C*H* = CHC_6_H_4_(p-OCH_3_)), 7.55 (d, *J* = 8.8 Hz, 2H, Ar*H*), 7.29 (t, *J* = 7.2 Hz, 3H, Ar*H*), 7.23–7.19 (m, 3H, Ar*H*), 6.97–6.93 (m, 2H, Ar*H*), 6.88–6.86 (m, 1H, Ar*H*), 6.66 (d, *J* = 2.0 Hz, 1H, Ar*H*), 4.51(t, *J* = 7.8 Hz, 2H, NC*H*_2_CH_2_CH_2_C_6_H_5_), 3.99 (t, *J* = 6.4 Hz, 2H, OC*H*_2_CH_2_CH_2_CH_3_), 3.86 (s, 3H, OC*H*_3_), 2.78 (t, *J* = 7.6 Hz, 2H, NCH_2_CH_2_C*H*_2_C_6_H_5_), 2.35–2.28 (m, 2H, NCH_2_C*H*_2_CH_2_C_6_H_5_), 1.87–1.80 (m, 2H, OCH_2_C*H*_2_CH_2_CH_3_), 1.61–1.51 (m, 3H, OCH_2_CH_2_C*H*_2_CH_3_), 1.03 (t, *J* = 7.4 Hz, 3H, OCH_2_CH_2_CH_2_C*H*_3_); ^13^C NMR (100 MHz, CDCl_3_) *δ* 160.4, 159.5, 143.2, 140.5, 139.3, 138.7, 134.0, 133.5, 130.4, 129.9, 128.4, 128.2, 128.1, 126.1, 122.0, 121.4, 114.5, 114.1, 112.3, 109.2, 93.4, 67.8, 55.1, 44.8, 32.9, 31.2, 31.1, 19.2, 13.8; HRMS (ESI) calcd for C_33_H_34_N_2_O_2_ [M + H]^+^ 491.2693, found 491.2703.

#### 1-(1-(4-Nitrostyryl))-9-acetyl-β-carboline(2g)

Yellow solid was obtained (2 g, 56%). Mp 200–201 °C; ESI-MS m/z: 358 [M+H]^+^; ^1^H NMR (400 MHz, CDCl_3_) *δ* 8.68 (s, 1H, Ar*H*), 8.54 (d, *J* = 5.2 Hz, 1H, Ar*H*), 8.24 (d, *J* = 8.8 Hz, 2H, Ar*H*), 8.15 (d, *J* = 8.0 Hz, 1H, Ar*H*), 7.97–7.93 (m, 2H, C*H* = C*H*C_6_H_4_(NO_2_)), 7.76 (d, *J* = 8.8 Hz, 2H, C*H* = C*H*C_6_H_4_(p-NO_2_)), 7.72–7.68 (m, 1H, Ar*H*), 7.63–7.58 (m, 2H, Ar*H*), 7.37–7.33 (m, 1H, Ar*H*); ^13^C NMR (100 MHz, DMSO-*d*_*6*_) *δ* 147.0, 144.2, 141.0, 139.0, 138.4, 135.2, 129.5, 129.4, 129.0, 128.4, 128.3, 124.6, 122.4, 121.3, 120.1, 115.2, 112.4; HRMS (ESI) calcd for C_21_H_15_N_3_O_3_ [M + H]^+^ 358.1186, found 358.1195.

#### 1-(4-Nitrostyryl)-9-(3-phenylpropyl)-β-carboline (2h)

Yellow solid was obtained (2.8 g, 65%). Mp 179–180 °C; ESI-MS m/z: 434 [M+H]^+^; ^1^H NMR (400 MHz, CDCl_3_) *δ* 8.51 (d, *J* = 4.8 Hz, 1H, Ar*H*), 8.26 (d, *J* = 8.8 Hz, 2H, Ar*H*), 8.15 (d, *J* = 7.6 Hz, 1H, Ar*H*), 7.95 (d, *J* = 4.8 Hz, 1H, Ar*H*), 7.91 (d, *J* = 2.8 Hz, 2H, Ar*H*), 7.66 (d, *J* = 8.8 Hz, 2H, Ar*H*), 7.60 (t, *J* = 7.6 Hz, 1H, Ar*H*), 7.38–7.29 (m, 4H, Ar*H*, CH = C*H*C_6_H_4_(p-OCH_3_)), 7.23 (d, *J* = 7.2 Hz, 1H, C*H* = CHC_6_H_4_(p-OCH_3_)), 7.16 (d, *J* = 6.8 Hz, 2H, Ar*H*), 4.61 (t, *J* = 7.6 Hz, 2H, NC*H*_2_CH_2_CH_2_C_6_H_5_), 2.79 (t, *J* = 7.2 Hz, 2H, NCH_2_CH_2_C*H*_2_C_6_H_5_), 2.37–2.29 (m, 2H, NCH_2_C*H*_2_CH_2_C_6_H_5_); ^13^C NMR (100 MHz, CDCl_3_) *δ* 147.0, 143.4, 141.9, 140.2, 139.0, 138.3, 134.5, 131.6, 130.9, 128.7, 128.6, 128.2, 128.0, 127.2, 126.4, 124.2, 121.5, 121.1, 120.2, 114.5, 109.6, 45.3, 33.3, 31.3; HRMS (ESI) calcd for C_28_H_23_N_3_O_2_ [M+H]^+^ 434.1863, found 434.1871.

#### 1-(4-Nitrostyryl)-7-butoxy-9-(3-phenylpropyl)-β-carboline (2i)

Yellow solid was obtained (3.2 g, 63%). Mp 170–171 °C; ESI-MSm/z: 506 [M + H]^+^; ^1^H NMR (400 MHz, CDCl_3_) *δ* 8.45 (d, *J* = 4.8 Hz, 1H, Ar*H*), 8.27–8.24 (m, 2H, Ar*H*), 7.97 (d, *J* = 8.4 Hz, 1H, Ar*H*), 7.89 (d, *J* = 1.2 Hz, 2H, Ar*H*), 7.82 (d, *J* = 4.8 Hz, 1H, Ar*H*), 7.67–7.64 (m, 2H, Ar*H*, CH = C*H*C_6_H_4_(p-NO_2_)), 7.32–7.28 (m, 2H, Ar*H*), 7.24–7.18 (m, 3H, Ar*H*), 6.90 (dd, *J* = 8.8, 2.0 Hz, 1H, Ar*H*), 6.68 (d, *J* = 2.0 Hz, 1H, C*H* = CHC_6_H_4_(p-NO_2_)), 4.50 (t, *J* = 7.8 Hz, 2H, NC*H*_2_CH_2_CH_2_C_6_H_5_), 4.00 (t, *J* = 6.4 Hz, 2H, OC*H*_2_CH_2_CH_2_CH_3_), 2.79 (t, *J* = 7.4 Hz, 2H, NCH_2_CH_2_C*H*_2_C_6_H_5_), 2.35–2.28 (m, 2H, NCH_2_C*H*_2_CH_2_C_6_H_5_), 1.88–1.81 (m, 2H, OCH_2_C*H*_2_CH_2_CH_3_), 1.58–1.52 (m, 2H, OCH_2_CH_2_C*H*_2_CH_3_), 1.03 (t, *J* = 7.4 Hz, 3H, OCH_2_CH_2_CH_2_C*H*_3_); ^13^C NMR (100 MHz, CDCl_3_) *δ* 160.8, 146.8, 143.5, 140.3, 139.1, 137.5, 134.6, 131.2, 131.1, 128.6, 128.2, 128.0, 127.1, 126.4, 124.1, 122.3, 114.5, 113.6, 109.6, 93.7, 68.0, 45.1, 33.0, 31.3, 31.0, 19.3, 13.9; HRMS (ESI) calcd for C_32_H_31_N_3_O_3_ [M+H]^+^ 506.2438, found 506.2446.

#### 1-(3,4,5-Trimethoxystyryl)-9-(3-phenylpropyl)-β-carboline (2j)

Yellow solid was obtained (0.36 g, 12%). Mp 145.0–145.2 °C; ESI-MS m/z: 479[M + H]^+^; ^1^H-NMR (400 MHz, DMSO-*d*_*6*_) *δ*: 8.21–8.23 (1H, d, *J* = 7.5Hz, Ar*H*), 7.84–7.86 (1H, d, *J* = 10.0Hz, Ar*H*), 7.61–7.62 (1H, d, *J* = 6.0 Hz, Ar*H*), 7.43–7.53 (2H, m, Ar*H*), 7.27–7.29 (1H, m, Ar*H*), 6.86–7.05 (7H, m, Ar*H*), 6.61 (2H, m, *CH* = *CH*), 4.31–4.35 (2H, t, J = 10.0Hz, NC*H*_2_CH_2_CH_2_Ph), 3.65 (3H, s, OC*H*_3_), 3.63 (6H, s, OC*H*_3_), 2.51–2.55 (2H, m, NCH_2_CH_2_C*H*_2_Ph), 2.07–2.09 (2H, m, NCH_2_C*H*_2_CH_2_Ph); ^13^C NMR (100 MHz, DMSO-*d*_*6*_) *δ* 153.1, 141.4, 139.9, 139.2, 138.4, 138.1, 133.9, 133.6, 132.5, 130.0, 128.2, 128.0, 127.7, 126.0, 122.8, 121.1, 120.7, 119.5, 113.2, 109.1, 103.7, 60.6, 55.6, 44.7, 32.8, 31.0; HRMS (ESI) calcd for C_31_H_30_N_2_O_3_ [M+H]^+^ 479.2329, found 479.2337.

### General synthetic procedure for the preparation of 1-benzylidine β-carbolines 3a–g

A mixture of 2 (2 mmol) and benzyl bromide (10–20 mmol) in ethyl acetate (50 ml) was refluxed for 8–20 hours. After completion of the reaction as indicated by TLC, the solution was cooled and filtered to afford yellow solid. The solid was recrystallized from ethanol to afford compounds 3a–g in 44–76% yields.

#### 1-(4-Methoxystyryl)-2-benzyl-β-carbolinium bromide (3a)

Yellow solid was obtained (0.45 g, 48%). Mp 147.6–148.9 °C; ESI-MS m/z: 391[M-Br]^+^; ^1^H NMR (400 MHz, DMSO-*d*_*6*_) *δ* 8.87 (d, *J* = 6.8 Hz, 1H, Ar*H*), 8.76 (d, *J* = 6.4 Hz, 1H, Ar*H*), 8.52 (d, *J* = 8.4 Hz, 1H, Ar*H*), 7.81 (d, *J* = 4.4 Hz, 2H, Ar*H*), 7.77 (d, *J* = 9.2 Hz, 2H, Ar*H*), 7.62 (d, *J* = 3.2 Hz, 2H), 7.50–7.46 (m, 1H, Ar*H*), 7.37–7.28 (m, 6H, Ar*H*, C*H* = C*H*C_6_H_4_(p-OCH_3_)), 7.09 (d, *J* = 8.8 Hz, 2H, Ar*H*), 6.14 (s, 2H, N^+^C*H*_2_C_6_H_5_), 3.85 (s, 3H, OC*H*_3_). ^13^C NMR (100 MHz, DMSO-*d*_*6*_) *δ* 161.6, 144.7, 143.9, 139.4, 135.6, 135.0, 134.1, 132.8, 132.1, 130.6, 129.4, 128.9, 128.2, 127.7, 123.8, 122.2, 120.2, 116.4, 114.8, 114.0, 113.7, 60.2, 55.9. HRMS (ESI) calcd for C_27_H_23_N_2_OBr [M-Br]^+^ 391.1805, found 391.1808.

#### 1-(4-Methoxystyryl)-2-benzyl-9-butyl-β-carbolinium bromide (3b)

Yellow solid was obtained (0.49 g, 46%).Mp 197–198 °C; ESI-MS m/z: 447[M-Br]^+^; ^1^H NMR (400 MHz, DMSO-*d*_*6*_) *δ* 8.99 (d, *J* = 6.4 Hz, 1H, Ar*H*), 8.92 (d, *J* = 6.4 Hz, 1H, Ar*H*), 8.60 (d, *J* = 8.0 Hz, 1H, Ar*H*), 7.98 (d, *J* = 8.4 Hz, 1H, Ar*H*), 7.91–7.87 (m, 1H, Ar*H*), 7.64–7.60 (m, 3H, Ar*H*, C*H* = CHC_6_H_4_(p-OCH_3_)), 7.54 (t, *J* = 7.2 Hz, 1H, Ar*H*), 7.35–7.33 (m, 3H, Ar*H*), 7.21–7.19 (m, 2H, Ar*H*), 7.06–7.01 (m, 3H, Ar*H*, CH=C*H*C_6_H_4_(p-OCH_3_)), 6.07 (s, 2H, N^+^C*H*_2_C_6_H_5_), 4.53 (t, *J* = 7.6 Hz, 2H, NC*H*_2_CH_2_CH_2_CH_3_), 3.83 (s, 3H, OC*H*_3_), 1.60–1.52 (m, 2H, NCH_2_C*H*_2_CH_2_CH_3_), 1.04–0.96 (m, 2H, NCH_2_CH_2_C*H*_2_CH_3_), 0.64 (t, *J* = 7.2 Hz, 3H, NCH_2_CH_2_CH_2_C*H*_3_). ^13^C NMR (100 MHz, DMSO-*d*_*6*_) *δ* 161.2, 145.6, 142.6, 140.0, 135.5, 135.4, 134.7, 134.3, 132.8, 129.7, 129.3, 128.8, 127.8, 127.5, 124.1, 122.6, 119.4, 117.0, 114.9, 113.4, 112.2, 60.4, 55.8, 30.9, 16.8, 13.8. HRMS (ESI) calcd for C_31_H_31_N_2_OBr [M-Br]^+^ 447.2431, found 447.2435.

#### 1-(4-Methoxystyryl)-2-benzyl-9-(3-phenylpropyl)-β-carbolinium bromide (3c)

Yellow solid was obtained (0.71 g, 60%). Mp 207–209 °C; ESI-MSm/z:509[M-Br]^+^; ^1^H NMR (400 MHz, DMSO-*d*_*6*_) *δ* 9.02 (d, *J* = 6.8 Hz, 1H, Ar*H*), 8.92 (d, *J* = 6.4 Hz, 1H, Ar*H*), 8.60 (d, *J* = 8.0 Hz, 1H, Ar*H*), 7.97 (d, *J* = 8.4 Hz, 1H, Ar*H*), 7.89 (t, *J* = 7.6 Hz, 1H, Ar*H*), 7.68–7.62 (m, 3H, Ar*H*), 7.54 (t, *J* = 7.6 Hz, 1H, Ar*H*), 7.36–7.34 (m, 3H, Ar*H*), 7.23–7.21 (m, 2H, Ar*H*), 7.13–7.04 (m, 6H, Ar*H*, C*H* = C*H*C_6_H_4_(p-OCH_3_)), 6.84–6.82 (m, 2H, Ar*H*), 6.09 (s, 2H,. N^+^C*H*_2_C_6_H_5_), 4.61 (t, *J*  =  7.6 Hz, 2H, NC*H*_2_CH_2_CH_2_C_6_H_5_), 3.84 (s, 3H, OC*H*_3_), 2.32–2.24 (m, 2H, NCH_2_CH_2_C*H*_2_C_6_H_5_), 1.93–1.85 (m, 2H, NCH_2_C*H*_2_CH_2_C_6_H_5_). ^13^C NMR (100 MHz, DMSO-*d*_*6*_) *δ* 160.7, 144.9, 142.0, 140.3, 139.3, 134.8, 134.2, 133.6, 132.0, 129.2, 128.6, 128.2, 127.9, 127.7, 127.2, 126.9, 125.6, 123.6, 123.3, 121.9, 118.9, 116.3, 114.4, 113.0, 111.5, 59.8, 55.3, 44.5, 31.9, 29.9. HRMS (ESI) calcd for C_36_H_33_N_2_OBr [M-Br]^+^ 509.2587, found 509.2596.

#### 1-(4-methoxystyryl)-2-benzyl-7-butoxy-9-(3-phenylpropyl)-β-carbolinium bromide (3d)

Yellow solid was obtained (0.93 g, 70%). Mp 214–215 °C; ESI-MS m/z: 581[M-Br]^+^; ^1^H NMR (400 MHz, DMSO-*d*_*6*_) *δ* 8.88 (d, *J* = 6.8 Hz, 1H, Ar*H*), 8.69 (d, *J* = 6.8 Hz, 1H, Ar*H*), 8.42 (d, *J* = 8.8 Hz, 1H, Ar*H*), 7.59 (d, *J* = 8.8 Hz, 2H, Ar*H*), 7.55 (d, *J* = 16.8 Hz, 1H, CH = C*H*C_6_H_4_(p-OCH_3_)), 7.37–7.28 (m, 4H, Ar*H*), 7.20–7.09 (m, 6H, Ar*H*), 7.06 (d, *J* = 8.8 Hz, 2H, Ar*H*), 7.01 (d, *J* = 16.8 Hz, 1H, CH = C*H*C_6_H_4_(p-OCH_3_)), 6.88–6.84 (m, 2H, Ar*H*), 5.98 (s, 2H, N^+^C*H*_2_C_6_H_5_), 4.54 (t, *J* = 7.8 Hz, 2H, NC*H*_2_CH_2_CH_2_C_6_H_5_), 4.18 (t, *J* = 6.4 Hz, 2H, OC*H*_2_CH_2_CH_2_CH_3_), 3.83 (s, 3H, OC*H*_3_), 2.28 (t, *J* = 7.5 Hz, 2H, NCH_2_C*H*_2_CH_2_C_6_H_5_), 1.90–1.83 (m, 2H m, 2H, NCH_2_C*H*_2_CH_2_C_6_H_5_), 1.82–1.76 (m, 2H, OCH_2_C*H*_2_CH_2_CH_3_), 1.56–1.46 (m, 2H, OCH_2_CH_2_C*H*_2_CH_3_), 0.98 (t, *J* = 7.4 Hz, 3H, OCH_2_CH_2_CH_2_C*H*_3_). ^13^C NMR (100 MHz, DMSO-*d*_*6*_) *δ* 163.1, 160.7, 147.4, 141.8, 140.6, 138.1, 135.2, 135.1, 134.2, 134.0, 129.2, 128.8, 128.3, 128.1, 127.9, 127.2, 127.1, 125.8, 124.8, 114.9, 114.5, 113.3, 112.9, 112.6, 94.3, 68.2, 59.5, 55.4, 44.2, 31.9, 30.6, 30.0, 18.8, 13.7. HRMS (ESI) calcd for C_40_H_41_N_2_O_2_Br [M+H]^+^ 581.3163, found 581.3170.

#### 1-(1-(4-Nitrostyryl)-2-benzyl-9-acetyl-β-carbolinium bromide (3e)

Yellow solid was obtained (0.80 g, 76%). Mp > 250 °C; ESI-MS m/z: 448[M-Br]^+^; ^1^H NMR (400 MHz, DMSO-*d*_*6*_) *δ* 9.25 (d, *J* = 6.4 Hz, 1H, Ar*H*), 9.03–8.97 (d, *J* = 6.4 Hz, 1H, Ar*H*), 8.64 (d, *J* = 7.6 Hz, 1H, Ar*H*), 8.31 (d, *J* = 8.8 Hz, 2H, Ar*H*), 8.16–8.12 (m, 1H, Ar*H*), 8.07 (d, *J* = 6.8 Hz, 1H, Ar*H*), 7.97–7.93 (m, 1H, Ar*H*), 7.86 (d, *J* = 8.8 Hz, 2H, Ar*H*), 7.70 (t, *J* = 7.2 Hz, 1H, Ar*H*), 7.30–7.25 (m, 5H, Ar*H*, CH = C*H*C_6_H_4_(p-NO_2_)), 7.12 (d, *J* = 16.8 Hz, 1H, C*H* = CHC_6_H_4_(p-NO_2_)), 6.17 (s, 2H, N^+^C*H*_2_C_6_H_5_), 2.66 (s, 3H, NCOC*H*_3_). ^13^C NMR (100 MHz, DMSO-*d*_*6*_) *δ* 171.0, 148.3, 143.1, 142.1, 141.2, 140.7, 139.5, 138.9, 135.2, 134.8, 134.1, 129.3, 129.1, 129.0, 128.0, 125.4, 124.6, 124.1, 121.9, 117.6, 115.4, 60.9, 28.2. HRMS (ESI) calcd for C_28_H_22_N_3_O_3_Br [M+H]^+^ 448.1656, found 448.1658.

#### 1-(4-Nitrostyryl)-2-benzyl-7-butoxy-9-(3-phenylpropyl)-β-carbolinium bromide (3f)

Yellow solid was obtained (0.89 g, 66%).Mp 228–229 °C; ESI-MS m/z: 596 [M-Br]^+^; ^1^H NMR (400 MHz, DMSO-*d*_*6*_) *δ* 8.93 (d, *J* = 6.8 Hz, 1H, Ar*H*), 8.75 (d, *J* = 6.4 Hz, 1H, Ar*H*), 8.44 (d, *J* = 8.8 Hz, 1H, Ar*H*), 8.33 (d, *J* = 8.8 Hz, 2H, Ar*H*), 7.99 (d, *J* = 16.8 Hz, 1H, C*H* = CHC_6_H_4_(p-NO_2_)), 7.87 (d, *J* = 8.8 Hz, 2H, Ar*H*), 7.34–7.30 (m, 4H, Ar*H*), 7.19–7.08 (m, 7H, Ar*H*, CH = C*H*C_6_H_4_(p-NO_2_)), 6.87–6.85 (m, 2H, Ar*H*), 6.01 (s, 2H, N^+^C*H*_2_C_6_H_5_), 4.51 (t, *J* = 7.6 Hz, 2H, NC*H*_2_CH_2_CH_2_C_6_H_5_), 4.18 (t, *J* = 6.4 Hz, 2H, OC*H*_2_CH_2_CH_2_CH_3_), 2.30 (t, *J* = 7.8 Hz, 2H, NCH_2_CH_2_C*H*_2_C_6_H_5_), 1.92–1.86 (m, 2H, NCH_2_C*H*_2_CH_2_C_6_H_5_), 1.83–1.76 (m, 2H, OCH_2_C*H*_2_CH_2_CH_3_), 1.56–1.46 (m, 2H, OCH_2_CH_2_C*H*_2_CH_3_), 0.98 (t, *J* = 7.4 Hz, 3H, OCH_2_CH_2_CH_2_C*H*_3_). ^13^C NMR (100 MHz, DMSO-*d*_*6*_) *δ* 163.8, 148.3, 148.1, 141.1, 141.0, 140.6, 137.0, 135.8, 135.6, 134.8, 134.6, 129.2, 128.9, 128.6, 128.3, 127.7, 126.3, 125.4, 124.6, 120.8, 115.8, 114.0, 113.1, 94.8, 68.7, 60.4, 44.8, 32.3, 31.1, 30.1, 19.2, 14.2. HRMS (ESI) calcd for C_39_H_38_N_3_O_3_Br [M-Br]^+^ 596.2908, found 596.2915.

#### 1-(3,4,5-Trimethoxystyryl)-2-benzyl-9-(3-phenylpropyl)-β-carbolinium bromide (3g)

Yellow solid was obtained (0.74 g, 57%). Mp 175–176 °C; ESI-MSm/z: 569 [M-Br]^+^; ^1^H-NMR (400MHz, DMSO-*d*_*6*_) *δ* 8.70–8.72 (1H, d, *J* = 8.0Hz, Ar*H*), 8.61–8.63 (1H, d, *J* = 7.5Hz, Ar*H*), 8.29–8.31 (1H, d, *J* = 10.0Hz, Ar*H*), 7.49–7.70 (3H, m, Ar*H*), 7.23–7.27 (1H, t, *J* = 9.0Hz, Ar*H*), 6.70–7.08 (11H, m, Ar*H*), 6.53–6.55 (2H, m, *CH* = *CH*), 5.81 (2H, s, N^+^C*H*_2_Ph), 4.31–4.35 (2H, t, *J* = 10.0Hz, NC*H*_2_CH_2_CH_2_Ph), 3.52 (6H, s, OC*H*_3_), 3.44 (3H, s, OC*H*_3_), 2.03–2.07 (2H, m, NCH_2_CH_2_C*H*_2_Ph), 1.63–1.65 (2H, m, NCH_2_C*H*_2_CH_2_Ph); ^13^C NMR (100 MHz, DMSO-*d*_*6*_) *δ* 153.3, 145.2, 142.5, 140.7, 139.3, 139.2, 135.1, 134.4, 133.9, 132.4, 130.1, 129.0, 128.5, 128.1, 127.9, 127.5, 125.9, 123.7, 122.3, 119.1, 116.7, 115.2, 111.8, 105.3, 60.2, 60.1, 56.2, 44.9, 32.2, 30.3; HRMS (ESI) calcd for C_38_H_37_N_2_O_3_Br [M-Br]^+^ 569.2799, found 569.2814.

### Cell culture and reagents

The human cancer cell lines were purchased from American Type Culture Collection (ATCC, Manassas, VA). The cell lines were maintained in McCoy’s 5A (for HCT116, RKO, DLD1 and HIEC cell lines), DMEM (for Hela, HepG2, LO2, HT29, A549, A375 and KB cell lines) and RPMI-1640 (for Bel-7402, BGC-823, 769-P and 786-0) supplemented with 10% FBS (Gibco) and 1% penicillin–streptomycin (Hyclone). The cells were incubated in an atmosphere of 5% CO_2 _at 37 °C. DMSO was obtained from Sigma Chemical Co. (St. Louis, MO, USA) PI3K pathway inhibitor, LY294002, was purchased from Cell Signaling Technology.

### Cell viability assay

Cells were seeded into 96-well flat-bottom plates at a density of 5 × 10^4 ^per well and cultured for 24 hours, then treated with increasing dosage of the tested compounds for the 24 hours. The cells viability was assayed using Cell Counting Kit-8 (CCK-8, Dojindo Laboratories, Kumamoto, Japan) following manufacturer’s protocol. The concentration required to inhibit cell growth by 50% (IC_50_) was calculated from viability curves as previously report^37^.

### CCK8 assay

The effect of the selected compound 3c on cell viability was determined by Dojindo Cell Counting Kit-8 (Dojindo Laboratories, Kumamoto, Japan).Cells were seeded at the density of 5 × 10^3^/well in 96-wells plate. After treated with compound 3c at the indicated concentrations or time points, cells were incubated with 10 μl of CCK-8 solution for 1 hour at 37 °C, according to the manufacturer’s instruction. Cell viability was then determined by a spectrophotometer set (ELx800, BioTek, USA) at a wavelength of 450 nm.

### Colony formation assay

HCT116 cells (1000 units) were counted and seeded in 6-wells plates for colony formation assay. Cells were cultured for 24 hours at 37 °C and then the media were replaced with media added with compound 3c at the indicated concentration. After 24 hours treatment, the media were changed to normal and cells were cultured for 10~14 days. The colonies were fixed with 4% paraformaldehyde (Electron Microscopy Sciences 16% Paraformaldehyde Cat.15700, diluted into PBS) for 30 minutes and stained with 0.1% Crystal Violet for 15 minutes and washed. The colonies were then photographed and counted by the Image J.

### DNA fragmentation assay

HCT116 cells cultured in 6-wells plates were treated with compound 3c at the indicated concentration for 24 hours. Cells were harvested and washed with PBS, then lysed with 20 μl lysis buffer (20 mM EDTA, 100 mM Tris, pH 8.0, and 0.8% SDS (w/v)). The cell lysates were supplemented with RNase A (0.5 mg/ml) and incubated at 37 °C for 1 hour. After added 10 μl proteinase K (20 mg/ml) and incubated for another 1 hour, the cell lysates were subjected to 1.5% agarose gel electrophoresis at 50 V for 1 hour. The DNA was visualized and photographed under ultraviolet illumination.

### Flow cytometry assay

For apoptosis analysis, 1 × 10^5 ^cells were collected from each sample and processed with Annexin V-FITC and PI (FITC Annexin V Apoptosis Detection Kit I (BD, CA, USA)) according to the manufacturer’s instructions. Briefly, HCT116 cells (1 × 105) were collected, washed with ice-cold PBS and resuspended in 100 μl binding buffer. Then, 2 μl of Annexin V-FITC and 5 μl of PI were added to the cells. After incubated for 15 minutes at room temperature in the dark, the cells were added an additional 400 μl of binding buffer and subjected to FACS cytometry. The results were analyzed with Summit version 4.3 software.

### ROS measurement

The oxidative stress of cells was determined by detection of cellular ROS with 5-(and-6)-carboxy-2′, 7′-dichlorodihydrofluorescein diacetate (carboxy-H_2_DCFDA; Invitrogen). HCT116 cells were collected, washed twice with PBS and resuspended in 1 ml PBS containing 10 μM H_2_DCFDA and incubated at room temperature for 30 minutes. Cells were then washed with PBS and the fluorescence intensities were measured by flow cytometry. The data were analyzed with Summit version 4.3 software.

### Luciferase assay

For luciferase assay, signaling pathway reporters were purchased from QIAGEN. (Cignal 45-Pathway Reporter Array, QIAGEN, Valencia, CA). The reporters and internal control were co-transfected into HCT116 cells for 24 h, cells were treated with 4 μM of compound 3c for another 24 h, and then the luciferase activity of firefly and Renilla cells was measured by dual Luciferase Reporter Assay System (Promega).

### Quantitative PCR

Total RNA was extracted using RNiso Plus (Takara, Dalian, China). First-stand cDNA was generated using the ReverAid first Strand cDNA Synthesis Kit (Thermo,MA,USA) with OligodT primer. The cDNAs were amplified by real-time PCR with Power SYBR Green PCR Master Mix (ABI 4367659) according to the manufacturer’s instructions. GAPDH mRNA level was used as internal normalization control. The primers used for PCR were GAPDH, 5′-TGCACCACCAACTGCTTAGC-3′ and 5′-GCATGGACTGTGGTCATGAG-3′; BAX, 5′-GAGGATGATTGCCGCCGTGGACA-3′ and 5′-GGTGGGGGAGGAGGCTTGAGG-3′; and Bcl-2, 5′-ATGTGTGTGGAGAGCGTCAACC-3′ and 5′-TGAGCAGAGTC TTCAGAGACAGCC-3′.

### Western blotting

Total cell lysates were harvested by RIPA buffer (1% v/v NP40, 0.5% w/v sodium deoxycholate, 0.1% w/v SDS) containing complete protease inhibitors (Roche Applied Sciences). The protein concentration was determined using the Pierce® BCA Protein Assay Kit (Pierce, 23225). Identical quantities of proteins were separated by 12% SDS-PAGE and transferred to PVDF membranes (PVDF, Millipore, cat # IPVH00010, Merck KGaA, Darmstadt, Germany). The membrane were blocked with 5% non-fat milk and the probed with indicated primary antibodies overnight at 4 °C. After incubated with specific horseradish peroxidase conjugated secondary antibodies, the bands were revealed by ECL detection system (Millipore). Antibodies used in this study are as follows: the primary antibodies, PARP (1:1000), caspase-8 (1:1000), caspase-9 (1:1000), caspase-3 (1:1000), cleaved-caspase-3 (1:1000), BAX (1:1000), Bcl-2 (1:1000), AKT (1:1000), pAKT (S-473) (1:1000), and ERK (1:1000), pERK (S-473) (1:1000) and GAPDH (1:5000) were all purchased from Cell Signaling Technology. HRP conjugated secondary antibodies, Pierce(R) Goat Anti-Rabbit IgG, (H&L) (#31460) and Pierce(R) Goat Anti-Mouse IgG, (H&L) (#31430) were purchased from Thermo.

### Xenograft tumor model

All experimental procedures involving animals were performed in accordance with the Guide for the Care and Use of Laboratory Animals (NIH publications Nos 80–23, revised 1996) and were approved by the Animal Care and Use Committee of Wuhan University. Six-week-old male BALB/c nude mice were purchased from HFK Technology Company (Beijing HFK Bioscience Co., Ltd) and used for xenograft tumor model. The experiments were carried out using HCT116 according to a previous report^38^. In brief, HCT116 cells (1 × 10^7^) were collected and suspended in 0.2 ml of PBS. 100 μl cells were injected subcutaneously (s.c.) into the lower flank of the mice. After seven days, mice bearing tumors of 50–100 mm^3^ were randomly divided into two groups (n = 10) and given the treatment (100 μl DMSO or 50 mg/kg body weight of compound 3c) every other day. Tumor diameters are measured with digital calipers every three days, and the tumor volume in mm^3 ^is calculated by the formula: Volume = (width)^2^ × length/2.

### Statistical analysis

The significance of the difference between different groups was determined with the Student’s *t* test and *P* values below 0.05 were considered statistically significant.

## Additional Information

**How to cite this article**: Zhang, X.-F. *et al*. Synthesis and mechanisms of action of novel harmine derivatives as potential antitumor agents. *Sci. Rep*. **6**, 33204; doi: 10.1038/srep33204 (2016).

## Supplementary Material

Supplementary Information

## Figures and Tables

**Table 1:** Cytotoxic activities of β-carboline derivatives *in vitro* (IC_50_, μM^a^).

Cmpd	Hela^b^	Bel-7402	HepG2	BGC-823	A549	A375	HT-29	769-P	786-0	KB	DLD1	HCT116	RKO
2a	139	190	171	>200	36.7	71.4	62.9	7.83	37.5	>200	>200	56.1	74.2
2b	142	118	60.5	167	47.1	42.2	21.3	25.6	12.9	29.5	>200	136	89.6
2c	50.5	34.6	32.2	66.2	12.8	23.9	27.8	5.72	14.4	24.6	39.3	43.7	32.6
2d	88.8	29.7	66.0	141	38.1	59.5	>200	25.2	67.4	30.1	30.8	31.5	28.5
2e	>200	>200	186	>200	127	>200	>200	123	92.3	58.9	95.2	123	78.6
2f	92.3	>200	92.9	>200	78.6	4.32	54.3	62.9	89.1	150	170	63.5	126
2g	>200	>200	197	>200	>200	>200	>200	>200	>200	>200	>200	>200	>200
2h	>200	>200	>200	>200	>200	>200	>200	>200	>200	>200	>200	>200	>200
2i	>200	>200	>200	>200	>200	>200	>200	>200	>200	>200	>200	>200	>200
2j	89.6	62.8	75.3	96.4	136	125	54.8	114	63.5	87.6	123	69.2	78.6
3a	32.1	22.5	9.63	22.9	10.8	28.2	9.41	4.90	10.0	17.9	30.8	8.52	5.40
3b	6.74	7.72	6.71	21.8	9.12	8.43	7.81	5.12	11.2	3.93	9.74	1.71	2.93
3c	4.52	2.92	2.11	0.46	3.93	0.68	4.32	4.61	3.22	0.93	1.63	3.82	3.21
3d	6.81	1.64	8.92	7.12	3.42	7.31	3.20	8.22	4.61	2.71	7.94	1.62	4.24
3e	14.4	17.9	5.23	14.8	7.62	25.3	10.5	19.8	28.6	15.4	21.2	5.70	4.52
3f	2.90	1.53	1.31	4.10	2.83	4.52	2.51	3.12	2.73	2.41	6.92	1.52	2.10
3g	3.20	2.14	3.42	8.02	3.03	2.92	8.24	2.53	1.72	7.94	6.80	2.62	2.63
Cisplatin	6.4	12.8	13.6	13.2	6.84	9.46	25.4	16.8	11.2	8.62	12.6	6.31	7.62

^a^Cytotoxicity as IC_50_ for each cell line, is the concentration of compound which reduced by 50% the optical density of treated cells with respect to untreated cells using the CCK8 assay.

^b^Cell line include (Hela), liver carcinoma (Bel-7402 and HepG2), gastric carcinoma (BGC-823), non-small cell lung carcinoma (A549), malignant melanoma (A375), colon carcinoma (HT-29), renal carcinoma (769-P and 786-0), epidermoid carcinoma of the nasopharynx (KB) and colorectal carcinoma (DLD, HCT116 and RKO).

^c^Data represent the mean values of three independent determinations.
