# Supplementary material for: Synthesis and mechanisms of action of novel harmine derivatives as potential antitumor agents
Source: Sci Rep. 2016 Sep 14;6:33204. doi: 10.1038/srep33204 (PMC5021947; doi:10.1038/srep33204)
Supplement: Supplementary Information [file srep33204-s1.doc]

**Synthesis and mechanisms of action of novel harmine derivatives as potential antitumor agents**

Xiao-Fei Zhang^3,2^, Rong-qin Sun^1^, Yi-fan Jia^4^, Qing Chen^1^, Rong-Fu Tu^2^, Ke-ke Li^2^, Xiao-Dong Zhang^2^, Run-Lei Du^2^,*, Ri-hui Cao^1^,*

^1^School of chemical and chemical engineering, Sun Yat-sen University, Guangzhou, 510275, P. R. China.

^2^College of Life Sciences, Wuhan University, Wuhan, 430072, P. R. China.

^3^School of Chemistry, Chemical Engineering and Life Sciences, Wuhan University of Technology, Wuhan, 430070, P. R. China.

^4^Renmin Hospital of Wuhan University, Hubei General Hospital, Wuhan, 430072, P. R. China.

*Correspondence to: Ri-hui Cao, PhD, Associate Professor; School of chemical and chemical engineering, Sun Yat-sen University, Guangzhou, 510275, P. R. China; Tel/Fax: 086-020-84110918/84112245; E-mail: caorihui@mail.sysu.edu.cn

Ri-hui Cao and Run-lei Du are both co-corresponding authors of this study.

**Supplemental data**

**
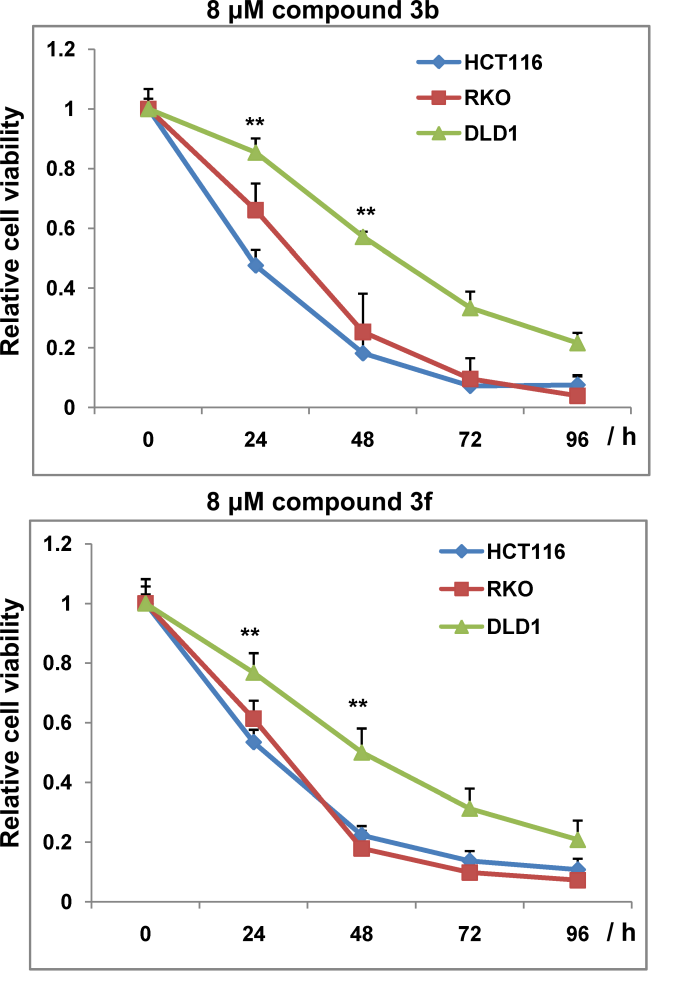
**

**Figure S1** The time-dependent inhibition effects of compound 3b and 3f on HCT116, RKO and DLD1 cells. Three cell lines were seeded in 96 well plates and treated with compound 3b (up panel) or 3f for the indicated time at the concentration of 8 μM (down panel). Cell viability was determined by CCK8 assays. (**P<0.05 versus OD value of HCT116 and RKO cells treated with the same concentration) The data represents the means ± SDs.


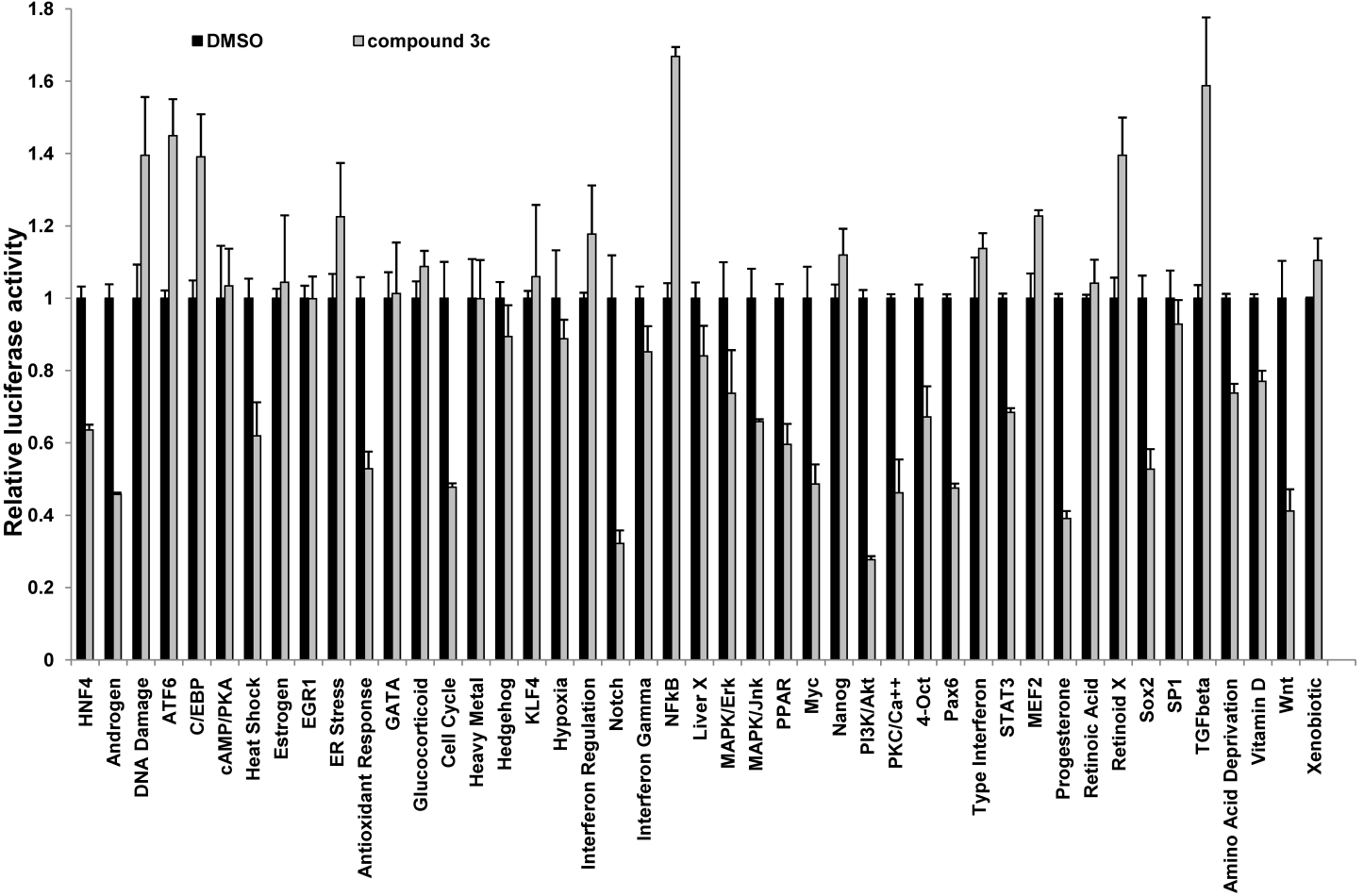


**Figure S2** Luciferase activity of 45-signaling pathway reporters. HCT116 cells were transfected with the indicated pathway reporter for 24 h, treated with 4 μM of compound 3c for another 24 h and then subjected to dual-luciferase assay. The data represents the means ± SDs.


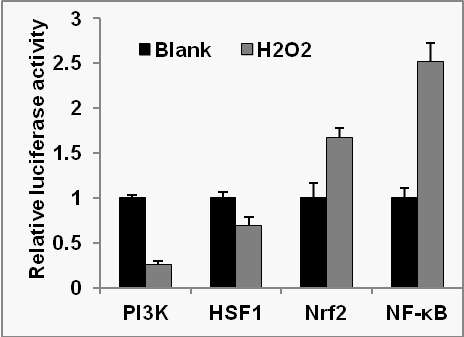


**Figure S3** The effect of H_2_O_2_ on reporter activities of HSF1, Nrf2 and NF-κb signaling. HCT116 cells were transfected with the indicated pathway reporter for 24 h, treated with 200 μM H_2_O_2_ for another 24 h and then subjected to dual-luciferase assay. The data represents the means ± SDs.

**
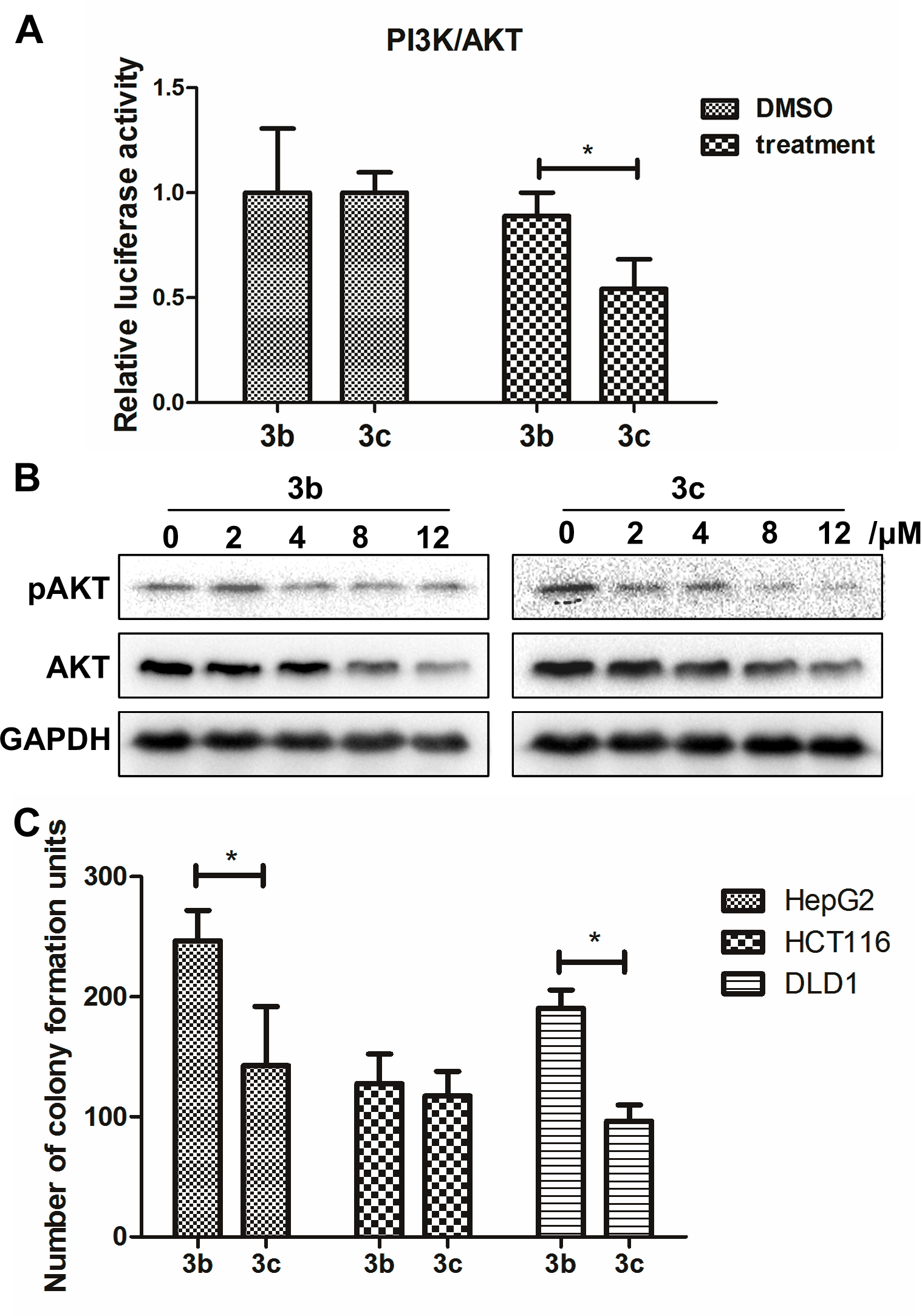
**

**Figure S4 Comparative analysis of the effect of compound 3b and 3c on PI3K/AKT signaling pathway and cell proliferation.** (A) Luciferase analysis of PI3K/AKT reporter activity. HCT116 cells were transfected with PI3K/AKT reporter for 24 hours and subjected to lucifease reader after 18 hours treatment with compound 3b or 3c. (n=3, *P<0.05 versus compound 3b treatment). (B) Protein levels of pAKT and total AKT following compound 3b or 3c treatment. HCT116 cells were treated with the indicated concentration of compound 3b or 3c for 72 h. Protein levels were detected by western blotting. (C) Colony formation assay of DLD1, HCT116 and HepG2. 500 cells were seeded in 12 well plates and treated with the 4 μM compound 3b or 3c for 12 h, then the media were replaced with complete culture media. After 8 days, the colony were stained  with [crystal violet](https://www.baidu.com/link?url=2TYQAIJlCovQTdmhhIo0zNymzOJawt0Zk8GXU0sM7Tw8d9BP6Yac-WudYrZMA5AWqrAxfEW-m7kLjHjUlhQMyp89RBlZB6M85rTrlwFZYvl3mkULaOyf8sOQJEl5xSwi&wd=&eqid=dce0980400035ddb00000003574e8547). The number of colony-forming units was counted with Image J. (n=3, *P<0.05 versus DMSO control). The data represents the means ± SDs.
